# Intratumoral androgen biosynthesis associated with 3β-hydroxysteroid dehydrogenase 1 promotes resistance to radiotherapy in prostate cancer

**DOI:** 10.1172/JCI165718

**Published:** 2023-11-15

**Authors:** Shinjini Ganguly, Zaeem Lone, Andrew Muskara, Jarrell Imamura, Aimalie Hardaway, Mona Patel, Mike Berk, Timothy D. Smile, Elai Davicioni, Kevin L. Stephans, Jay Ciezki, Christopher J. Weight, Shilpa Gupta, Chandana A. Reddy, Rahul D. Tendulkar, Abhishek A. Chakraborty, Eric A. Klein, Nima Sharifi, Omar Y. Mian

**Affiliations:** 1 Translational Hematology and Oncology Research,; 2 Thermo Fisher Scientific, Ann Arbor, Michigan, USA.; 3 Department of Cancer Biology, Lerner Research Institute,; 4 Department of Radiation Oncology, and Taussig Cancer Institute, Cleveland Clinic, Cleveland, Ohio, USA.; 5 Veracyte Inc., San Francisco, California, USA.; 6 Glickman Urologic and Kidney Institute, Cleveland Clinic, Cleveland, Ohio, USA.; 7 Desai Sethi Urology Institute and Sylvester Comprehensive Cancer Center, University of Miami Miller School of Medicine, Miami, Ohio, USA.

**Keywords:** Endocrinology, Therapeutics, Prostate cancer, Radiation therapy, Sex hormones

## Abstract

Half of all men with advanced prostate cancer (PCa) inherit at least 1 copy of an adrenal-permissive *HSD3B1* (1245C) allele, which increases levels of 3β-hydroxysteroid dehydrogenase 1 (3βHSD1) and promotes intracellular androgen biosynthesis. Germline inheritance of the adrenally permissive allele confers worse outcomes in men with advanced PCa. We investigated whether *HSD3B1* (1245C) drives resistance to combined androgen deprivation and radiotherapy. Adrenally permissive 3βHSD1 enhanced resistance to radiotherapy in PCa cell lines and xenograft models engineered to mimic the human adrenal/gonadal axis during androgen deprivation. The allele-specific effects on radiosensitivity were dependent on availability of DHEA, the substrate for 3βHSD1. In lines expressing the *HSD3B1* (1245C) allele, enhanced expression of DNA damage response (DDR) genes and more rapid DNA double-strand break (DSB) resolution were observed. A correlation between androgen receptor (AR) expression and increased DDR gene expression was confirmed in 680 radical prostatectomy specimens. Treatment with the nonsteroidal antiandrogen enzalutamide reversed the resistant phenotype of *HSD3B1* (1245C) PCa in vitro and in vivo. In conclusion, 3βHSD1 promotes prostate cancer resistance to combined androgen deprivation and radiotherapy by upregulating DNA DSB repair. This work supports prospective validation of early combined androgen blockade for high-risk men harboring the *HSD3B1* (1245C) allele.

## Graphical abstract

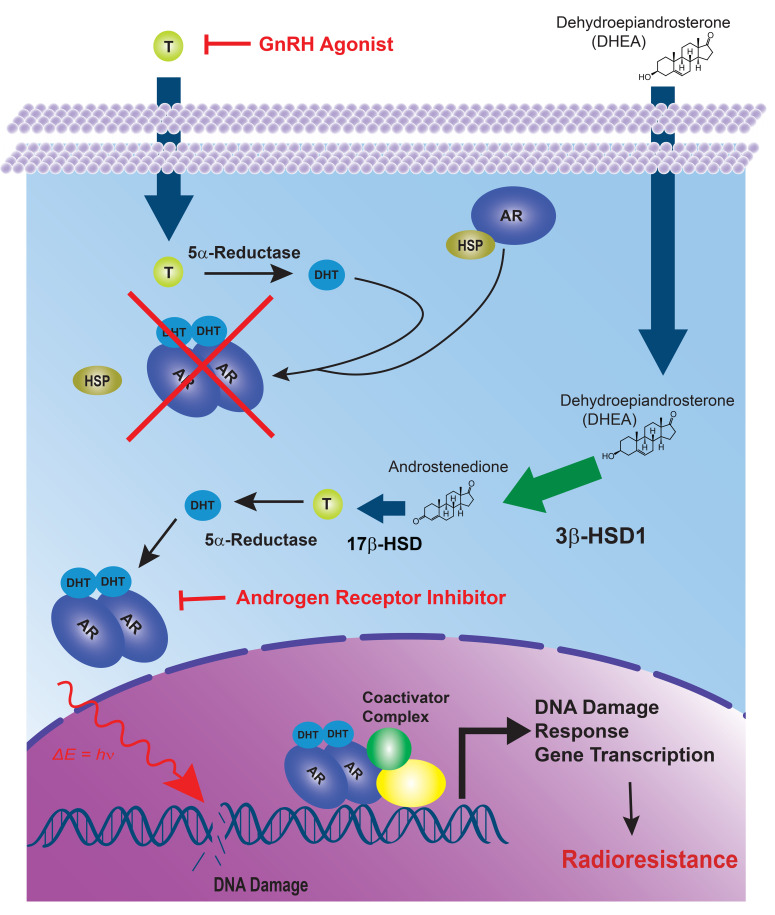

## Introduction

In men with high-grade prostate cancer (PCa) treated with curative-intent radiotherapy, the addition of androgen-deprivation therapy (ADT) confers a clear and sustained benefit in terms of local control, metastasis-free survival (MFS), and overall survival (OS) (1–3). This benefit is observed across unfavorable risk groups and is not precluded by radiotherapy dose escalation (4, 5). One mechanism for the observed combinatorial benefit of ADT and ionizing radiation (IR) is the androgen receptor’s (AR’s) direct activation of DNA damage response (DDR) gene transcription, promoting radiation-induced double-strand break (DSB) repair and radioresistance (6–8). In addition to centrally acting gonadotropin-releasing hormone (GnRH) agonists or antagonists (e.g., leuprolide, goserelin, and relugolix), treatment with AR inhibitors (ARIs) (e.g., bicalutamide, apalutamide, and enzalutamide [Enza]) (9–12) in combination with radiotherapy has been explored (13); however, comparative efficacy data are lacking. Similarly, biomarkers for tailored patient selection for combined radiation and androgen blockade with GnRH agonists and/or ARIs remain the subject of ongoing investigation (14–16).

PCa cells bypass the need for circulating testosterone (T) through a variety of mechanisms including tumor-intrinsic synthesis of T or dihydrotestosterone (DHT) from extragonadal steroid precursors, e.g., adrenal dehydroepiandrosterone (DHEA) (17, 18). This phenomenon in part drives the significant clinical benefit of combining ADT with ARIs or CYP17A inhibitors (e.g., abiraterone) in advanced and metastatic hormone-sensitive PCa (mHSPC) (19–22). The rate-limiting step in the metabolic conversion from DHEA to T in PCa is catalyzed by the enzyme 3β-hydroxysteroid dehydrogenase 1 (3βHSD1), encoded by the gene *HSD3B1* (17). A common germline polymorphism observed in *HSD3B1* results in a single amino acid substitution, asparagine to threonine, at amino acid position 367 (p.N367T). This substitution confers resistance to proteolytic degradation, leading to intracellular accumulation of the enzyme associated with the emergence of castration-resistant prostate cancer (CRPC) (17, 23, 24). The *HSD3B1* (1245C) allele is termed adrenal permissive, as it enables increased conversion of DHEA to more potent androgens, whereas the *HSD3B1*(1245A) allele is adrenal restrictive because it limits this metabolic flux. We previously reported that germline homozygosity for the adrenal-permissive 1245C allele was associated with the more rapid development of castrate-resistant disease in patients who relapsed after salvage radiotherapy and ADT (25). These findings led us to hypothesize that extragonadal (tumor cell intrinsic) synthesis of androgens from adrenal precursors by increased intracellular 3βHSD1 would modulate the PCa response to genotoxic stress. In the present work, we explore this clinically actionable relationship between *HSD3B1* genotype and PCa resistance to radiation therapy.

## Results

### The adrenal-permissive HSD3B1 (1245C) allele confers a proliferative advantage to prostate cells in a DHEA-dependent manner.

**Figure 1:**
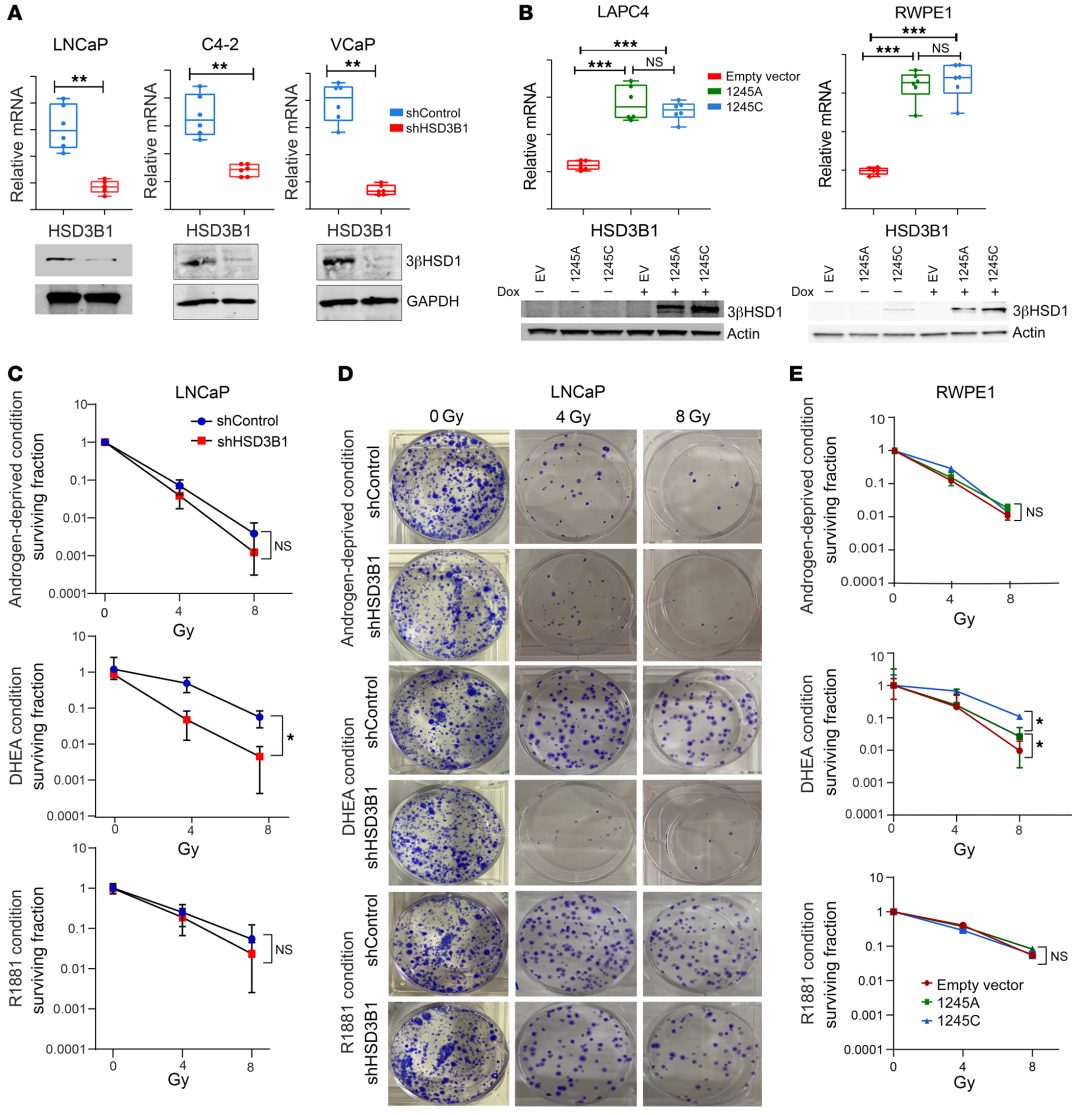
Loss of 3βHSD1 expression reduces colony formation and cell survival in irradiated PCa cells. *HSD3B1* mRNA (top panels) and 3βHSD1 protein expression (bottom panels) in (**A**) LNCaP, C4-2, and VCaP cells stably expressing shRNA targeting *HSD3B1* (shHSD3B1) or nonsilencing shRNA (shControl). Gene expression was normalized to *ACTB*, and GAPDH was used as a loading control for immunoblotting. All data are represented as mean ± 95% CI from triplicates of 2 independent experiments (unpaired 2-tailed *t* test) (**B**) LAPC4 and RWPE-1 cells stably expressing dox-inducible restrictive (1245A) or permissive (1245C) *HSD3B1*. Gene expression of HSD3B1 (top panels) was assessed by qPCR (normalized to *ACTB*). All data are represented as mean ± SEM from triplicates of 2 independent experiments (1-way ANOVA with Bonferroni’s multiple-comparison test), and protein levels of 3βHSD1 (bottom panels) were measured by immunoblotting (normalized to β-actin). (**C**) Surviving cell fraction and (**D**) colony formation assay of LNCaP cells expressing shHSD3B1 or shControl treated with 4 or 8 Gy radiation and cultured for 14 days in csFBS medium containing ethanol (top panel), 50 nM DHEA (middle panel), or 1 nM R1881 (bottom panel) followed by crystal violet staining. Representative images of colonies formed after treatment with 0, 4, or 8 Gy radiation and cultured in the presence or absence of androgens. Original magnification, ×2. (**E**) The number of viable RWPE1 colonies stably expressing 1245A or 1245C *HSD3B1* treated with 0, 4, or 8 Gy radiation and cultured in csFBS media containing ethanol (top panel), 50 nM DHEA (middle panel), or 1 nM R1881 (bottom panel). All data are represented as mean values ± 95% CI from triplicates in 2 independent experiments (unpaired 2 tailed *t* test). *P < 0.05; **P < 0.01; ***P < 0.001.

To explore the effect of intratumoral conversion of DHEA to DHT on the proliferative capacity of prostate cell lines harboring the adrenal-permissive *HSD3B1* allele (i.e., LNCaP, VCaP, and C4-2), the gene was stably knocked down using lentiviral shRNA targeting *HSD3B1*. shHSD3B1 transduction resulted in an average 81% transcript knockdown in LNCaP, 58% in C4-2, and 83% in VCaP cells (*P* < 0.001), assessed by quantitative real-time PCR (RT-qPCR). Enzyme knockdown was validated by immunoblotting with anti-3βHSD1 antibodies (Figure 1A). LNCaP short hairpin control (shControl) cells grew 2.2 times faster than shHSD3B1 cells when cultured in DHEA-supplemented media (*P* < 0.05) (Supplemental Figure 1A; supplemental material available online with this article; https://doi.org/10.1172/JCI165718DS1). The proliferative disadvantage observed for shHSD3B1 cells was completely rescued when the cells were treated with the synthetic androgen R1881. Under androgen-deprived conditions (charcoal-stripped FBS–containing [csFBS–containing) media), both shControl and shHSD3B1 cells proliferated at a similar diminished rate (Supplemental Figure 1A). Proliferation effects were confirmed using C4-2 (relative proliferation rate [RPR] = 1.3, *P* < 0.005) and VCaP cells (RPR = 2.5, *P* < 0.005) (Supplemental Figure 1, B and C).

The adrenal-permissive allele (1245C) or the restrictive allele (1245A) was individually overexpressed in LAPC4 and RWPE1 cells, which have low endogenous levels of the 3βHSD1 enzyme, using a doxycycline-inducible (dox-inducible) system. Although 1245A and 1245C showed comparable mRNA levels, the protein levels of the adrenal-permissive variant were higher (Figure 1B), consistent with the stabilizing effect of the 1245C polymorphism (17). When cells were supplemented with DHEA, the average proliferative rate of the adrenal-permissive allele-harboring LAPC4 cells was greater than that of cells transduced with either the restrictive allele (RPR= 1.3 *P* < 0.05) or empty vector (EV) (RPR = 2.0 *P* < 0.05) (Supplemental Figure 1D). Similar observations were made for RWPE1 cells, where populations transduced with the 1245C allele grew 1.9 times faster than 1245A allele–transduced cells and 4 times faster than EV-transduced cells (Supplemental Figure 1E). This difference was dependent on the presence of DHEA and was fully rescued by treatment with R1881 (Supplemental Figure 1, D and E). Both results were consistent with an increase in flux from DHEA to T, catalyzed by increased levels of 3βHSD1 in 1245C cells.

### The adrenal-permissive 1245C HSD3B1 allele drives DHEA-dependent radioresistance in both PCa cells and immortalized prostatic epithelial cells.

We next investigated whether intratumoral androgen biosynthesis was associated with differences in radiosensitivity in PCa cells. shControl and shHSD3B1 LNCaP cells were plated at clonogenic density and irradiated in androgen-depleted medium (10% csFBS media), DHEA-supplemented medium (50 nM DHEA), and androgen-supplemented medium (1 nM R1881). Under androgen-deprived conditions, both shControl and shHSD3B1 cells were similarly radiosensitive. When supplemented with R1881, both shHSD3B1 and shControl cells exhibited a radioresistant phenotype (SF8 = 5.4 × 10^–2^ and SF8 = 2.2 × 10^–2^, respectively, where SF8 indicates surviving fraction at 8 Gy; *P* > 0.05) (Figure 1, C and D). However, in DHEA medium, the surviving fractions following 4 Gy and 8 Gy radiation were significantly higher in shControl cells compared with shHSD3B1 (SF4 = 4.2 × 10^–1^ versus 4.1 × 10^–2^; SF8 = 4.9 × 10^–2^ versus 3.9 × 10^–3^, *P* < 0.005). Similar observations were made for C4-2 and VCaP cells (Supplemental Figure 2A). Next, the relationship between intratumoral androgen biosynthesis and radioresistance was explored in the context of localized, hormone-sensitive PCa. To this end, we used immortalized prostate epithelial cell line RWPE1. LAPC4 cells were not amenable to in vitro colony-forming assays, as they did not reliably disaggregate into single-cell suspensions and do not survive plating at clonogenic densities. In RWPE1 cells, increased radioresistance was observed in cells expressing the *HSD3B1* (1245C) allele compared with the *HSD3B1* (1245A) allele and EV-transduced control cells. *HSD3B1* (1245C) cells cultured in DHEA had a higher clonogenic survival capacity following treatment with 8 Gy IR, with a survival fraction (SF8 = 1.0 × 10^–1^) significantly greater than either *HSD3B1* (1245A) allele (SF8 = 2.6 × 10^–2^, *P* < 0.005) or EV-transduced (SF8 = 9.6 × 10^–3^, *P* < 0.0005) RWPE1 cells (Figure 1E).

### Differences in radiosensitivity were not attributable to cell-cycle redistribution.

Observed DHEA-dependent variability in clonogenic survival supported the hypothesis that the *HSD3B1* (1245C) allele promotes radioresistance by driving intracellular accumulation of androgens via increased conversion of DHEA, engaging the AR axis. Because radiosensitivity varies throughout the cell cycle, with bimodal peaks in the early S phase and mitosis (26), we investigated whether observed differences in radiosensitivity could be attributed to variable cell-cycle distribution. We performed cell-cycle analysis using LNCaP (shControl and shHSD3B1 cells) and RWPE1 cells (EV, 1245A, and 1245C) and found no systematic reassortment in cell-cycle distribution between the cell populations (Supplemental Figure 2B), indicating that differences in radiosensitivity were not attributable to cell-cycle differences.

### Increased 3βHSD1 levels drive radioresistance in vivo.

**Figure 2:**
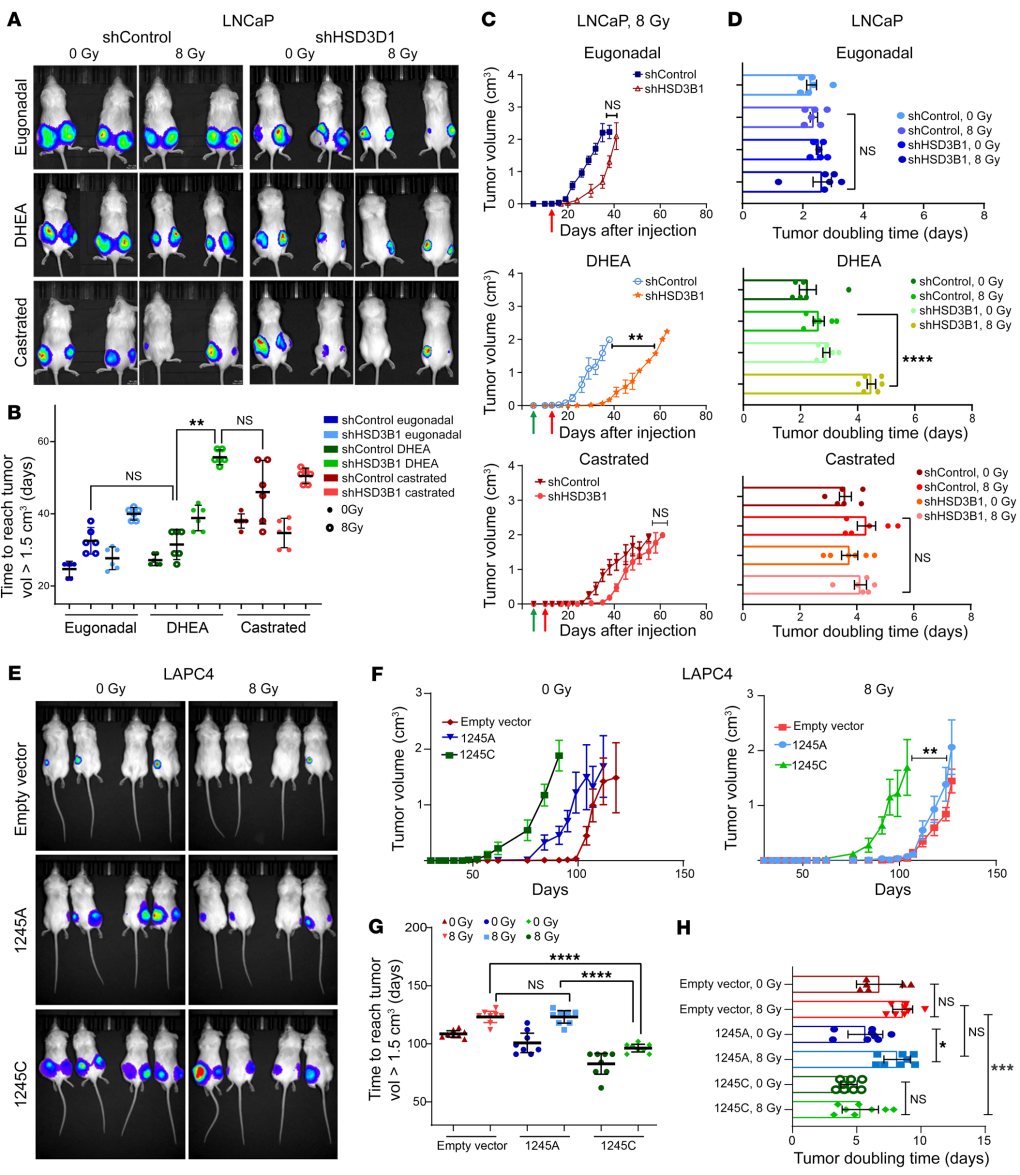
Loss of 3βHSD1 suppresses tumor growth of LNCaP cells following radiation treatment. (**A**) Representative bioluminescence images of LNCaP subcutaneous tumors expressing shRNA targeting *HSD3B1* (right panels) or a nontargeting control (left panels) grown in male NSG mice after radiation. Following subcutaneous injection of LNCaP cells, mice were divided into 3 groups: eugonadal (top panels), castrated with DHEA pellet supplement (middle), and castration alone (bottom). Mice from each group were sham irradiated or irradiated with 8 Gy and imaged when tumors reached 2.5 cm^3^. (**B**) The average number of days required for control (*n* = 6) and shHSD3B1 (*n* = 6) LNCaP tumors grown in eugonadal, castrated, and DHEA pellet–implanted mice to reach an end point size of 1.5 cm^3^. Data are represented as mean ± 95% CI (*P* values were calculated using 1-way ANOVA with Bonferroni’s multiple-comparison test). (**C**) Tumor growth of shControl and shHSD3B1 LNCaP xenograft tumors following radiation treatment in eugonadal (top), castrated (bottom), and DHEA-supplemented mice (middle). Red arrows, time of irradiation; green arrows, time of surgery. (**D**) Tumor-doubling time of irradiated shControl and shHSD3B1 LNCaP xenografts in eugonadal mice (top), castrated mice (bottom), and castrated mice with DHEA implantation (middle). Data are represented as mean ± SEM (*P* values were calculated using 1-way ANOVA with Bonferroni’s multiple comparison test).(**E**) Representative bioluminescence imaging and (**F**) tumor growth of LAPC4 subcutaneous tumors expressing 1245A, 1245C *HSD3B1*, or EV allele after sham (left panel, *n* = 8 per group) or 8 Gy radiation (right panel, *n* = 8 per group). (**G**) The number of days after irradiation for EV (*n* = 8), 1245A (*n* = 8), and 1245C (*n* = 8) *HSD3B1* LAPC4 tumors to reach an end point size of 1.5 cm^3^. Data are represented as mean ± 95% CI (*P* values were calculated using 1-way ANOVA with Bonferroni’s multiple-comparison test). (**H**) Tumor-doubling time of irradiated or sham-treated LAPC4 xenograft tumors expressing EV, 1245A, and 1245C allele. For **C** and **F**, data are represented as mean ± SEM. *P* values were calculated using Mann-Whitney *U* test for nonparametric data analysis. For **B**, **D**, **G**, and **H**, data are represented as mean ± 95% CI (*P* values were calculated using 1-way ANOVA with Bonferroni’s multiple-comparison test). *P < 0.05; **P < 0.01; ***P < 0.001; ****P < 0.0001.

The in vitro findings of radioresistance associated with *HSD3B1* transcript levels were confirmed in vivo using LNCaP shControl and shHSD3B1 xenografts, treated as described in Methods (see *Mouse xenograft studies*) (Figure 2A). Following radiation treatment, the median time for shControl tumors to reach a volume of 1.5 cc in castrate DHEA-implanted mice did not differ from times observed in intact eugonadal mice: 31.5 days (IQR: 27.3, 35.6) and 32.5 days (IQR: 28.8, 36.1), respectively. However, shHSD3B1 tumors implanted in castrate DHEA-implanted mice took an average of 55 days (IQR: 52, 57) to reach the same end point. The extended duration was similar to the observed longer intervals for shControl tumors in castrated mice, 46.5 days (IQR: 37.25, 55) (Figure 2B). Radiation treatment induced a significantly greater delay in tumor growth for shHSD3B1 tumors compared with that observed for shControl tumors in castrate DHEA pellet–implanted mice (Figure 2C). There was no significant difference in growth kinetics in castrated animals without DHEA pellet implantation, where both shControl and shHSD3B1 tumors grew relatively slowly. In sham-treated, intact eugonadal animals, both shControl and shHSD3B1 tumors grew rapidly following radiation (Figure 2C and Supplemental Figure 3, A and B). In fact, the shHSD3B1 xenografts matched the enhanced radiosensitivity observed in castrate mice while shControl xenografts exhibited radiation resistance comparable to that of tumors in intact eugonadal mice. Furthermore, following radiation, the mean tumor doubling time of shControl xenografts was significantly slower than that observed for shHSD3B1 tumors (2.57 versus 4.6 days) in DHEA pellet–implanted mice (Figure 2D). In summary, there was a significant difference in shControl and shHSD3B1 tumor growth after radiation under conditions mimicking human gonadal androgen axis suppression (ADT), where circulating adrenal precursors, e.g., DHEA, remained available. Therefore, the effect of 3βHSD1 levels on radiosensitivity in the DHEA-implanted mice abolished the radiosensitizing effect of castration.

### The adrenal-permissive HSD3B1 (1245C) allele drives radioresistance in vivo.

LAPC4 xenografts transduced with an EV control, *HSD3B1* (1245A) or *HSD3B1* (1245C), were established in the flanks of 8- to 12-week-old male mice that subsequently underwent surgical orchiectomy with subcutaneous implantation of a controlled continuous-release DHEA pellet. After flank-tumor irradiation, the 1245C allele–expressing LAPC4 tumors rebounded faster and reached the experimental end point sooner than the 1245A allele–expressing tumors. Both EV and 1245A tumors grew more slowly following radiation treatment than 1245C LAPC4 cells (Figure 2, E–H). Under in vivo conditions designed to model human adrenal physiology, adrenal-permissive *HSD3B1* (1245C) allele–expressing xenografts were more radioresistant, with minimal growth inhibition following a divided dose of 8 Gy, when compared with relatively radiosensitive adrenal restrictive 1245A- and EV-transduced LAPC4 tumors. Taken together, these in vitro and in vivo data consistently demonstrated that intratumoral accumulation of 3βHSD1 drives a radioresistant phenotype in a substrate-dependent (DHEA) manner.

### The adrenally permissive HSD3B1 (1245C) allele enhances IR-induced DDR in PCa and immortalized prostate epithelial cells.

**Figure 3:**
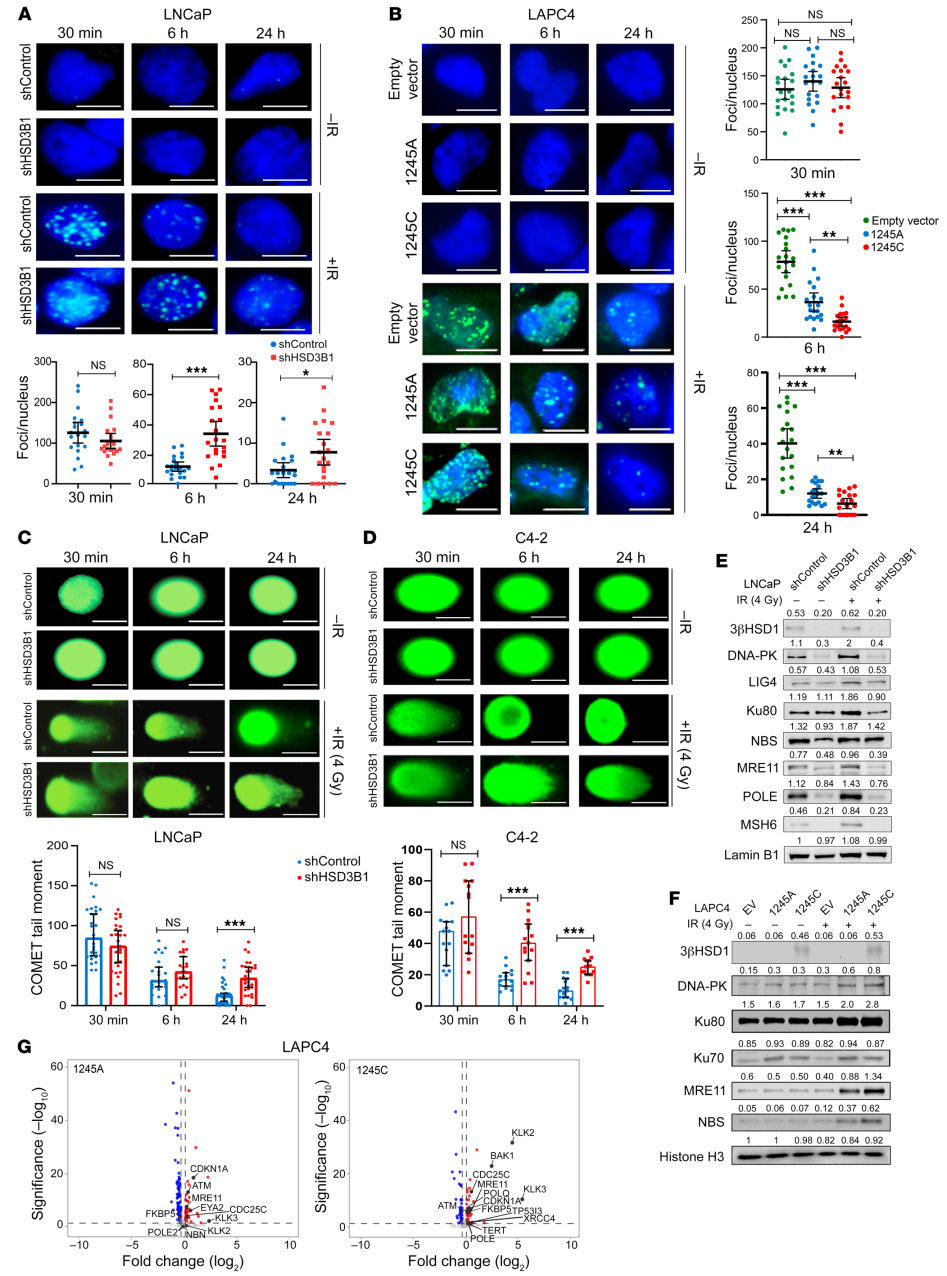
3βHSD1 enhances DNA repair in irradiated PCa cells treated with DHEA. (**A**) Representative immunofluorescence images (top) and quantitation (bottom) of phospho-γH2AX foci in LNCaP cells expressing shControl or shHSD3B1 pretreated with 50 nM DHEA for 48 hours followed by 0 Gy or 4 Gy irradiation. The γH2AX foci were quantified as foci per nucleus at each time point. All data are represented as mean values ± 95% CI (*P* values were calculated using 2-tailed *t* test). (**B**) Representative immunofluorescence images (top) and quantification (bottom) of γH2AX foci in LAPC4 cells stably expressing *HSD3B1* 1245A, 1245C, or EV control. The cells were treated for 48 hours with DHEA, followed by 0 Gy or 4 Gy irradiation. All data are represented as mean values ± 95% CI (*P* values were calculated using 2-way ANOVA with Bonferroni’s multiple comparison test). (**C**) Neutral COMET assay and tail moment quantitation of LNCaP and (**D**) C4-2 cells expressing shControl and shHSD3B1 following pretreatment with DHEA and irradiation (0 Gy and 4 Gy). All data are represented as mean values ± 95% CI (*P* values were calculated using 2-tailed *t* test) (47). *P < 0.05; **P < 0.01; ***P < 0.001. Scale bars: 5 μm. (**E**) Immunoblot of DDR markers 12 hours after irradiation in LNCaP cells pretreated with 50 nM DHEA. Lamin B1 was used as a loading control. (**F**) Immunoblot analysis of DDR markers 12 hours after irradiation in LAPC4 expressing *HSD3B1* (1245A, 1245C, or EV control) pretreated with 50 nM DHEA. (**G**) Volcano plots depicting differentially expressed genes in *HSD3B1* (1245A) LAPC4 cells (left) and *HSD3B1* (1245C) LAPC4 cells (right) compared with the EV control. Key DDR genes are highlighted in black.

To determine whether the increased radioresistance observed was a function of AR-driven upregulation of the DDR, LNCaP, LAPC4, and RWPE1 cells (immortalized epithelial cells derived from the peripheral zone of a histologically normal adult human prostate) were supplemented with 50 nM DHEA for 48 hours, followed by a single fraction of 4 Gy γ radiation. Phospho-γH2a.X foci were quantified at 30 minutes, 6 hours, and 24 hours after radiation treatment, subtracting the background foci in corresponding unirradiated samples (Figure 3, A and B, and Supplemental Figure 4, A–C). The initial γH2a.X foci counts, analogous to individual DNA DSBs, at 30 minutes were similar across populations. Comparing the break resolution kinetics in LNCaP cells over the following 24 hours, shHSD3B1 cells exhibited a significantly higher number of residual γH2a.X foci than observed for corresponding shControl cells at 6 and 24 hours after radiation (*P* < 0.05) (Figure 3A and Supplemental Figure 4A). This finding was consistent in LAPC4 and RWPE1 cells overexpressing the 1245C and 1245A alleles, where the adrenal-restrictive 1245A cells exhibited less efficient DNA DSB resolution following a single treatment of 4 Gy in the presence of DHEA compared with adrenal-permissive 1245C cells (Figure 3B and Supplemental Figure 4B). Neutral comet assays performed under the same conditions recapitulated the results obtained in γ-H2AX foci formation assays, showing that shControl cells had significantly smaller comet tail moments 24 hours after 4 Gy irradiation compared with shHSD3B1 LNCaP and C4-2 cells (Figure 3, C and D, and Supplemental Figure 4, D–F). Under neutral conditions, S-phase DNA contains replication bubbles that retard migration during electrophoresis (27). To account for cell-cycle effects and cell ploidy as confounders in the measurement of DHEA-mediated DNA DSB repair, bivariate plots of percentages of DNA in the tail (comet tail moment) versus total DNA content (comet intensity) were generated. No differences in the expected “horseshoe” distribution of LNCaP shControl and shHSD3B1cells were observed (Supplemental Figure 4E). These data show that, in the presence of DHEA, the 1245C allele–harboring cells more efficiently resolved IR-induced DNA damage.

### The adrenally permissive HSD3B1 (1245C) allele is associated with increased expression of DDR genes.

Given the known association between canonical AR output and DNA repair gene expression in primary PCas (8, 28, 29), the expression of DNA repair genes was measured in *HSD3B1* (1245C) cell lines to determine whether repair protein levels were correlated with observed differences in radiosensitivity. Initially, to account for the variable expression of repair proteins throughout the cell cycle, temporal kinetics experiments in synchronized populations were performed to establish basal patterns of DNA-damage protein expression. These experiments revealed that LNCaP and LAPC4 cells synchronously released from androgen deprivation by a 24-hour R1881 treatment followed by a single radiation dose of 4 Gy showed peak DDR protein induction 12 hours after IR exposure (Supplemental Figure 5A). However, this observation was not uniform, as some DDR proteins (e.g., Ku70) exhibited peak expression at earlier time points. For subsequent experiments, the peak expression time point for individual DDR proteins was used to consistently assess DDR induction. Following irradiation (4 Gy), LNCaP shControl–transduced cells showed significantly higher levels of DNA-PKc, Lig4, Ku80, MRE11, NBS, POLE, and MSH6 compared with shHSD3B1-transduced cells in DHEA media (Figure 3E). These results support the hypothesis that in the presence of DHEA, increased 3βHSD1, which is associated with the adrenally permissive *HSD3B1* allele, results in enhanced induction of DDR genes at baseline (0 Gy) and this effect is further amplified after irradiation (4 Gy). To validate the association of DDR genes and radioresistance, siRNA-mediated silencing of *PRKDC* in LNCaP cells was used. siPRKDC restored radiosensitivity in shControl cells that were otherwise resistant in DHEA-supplemented media. Silencing of *PRKDC* in shHSD3B1 cells did not have any additive radiosensitizing effect, consistent with basally low levels of DNA-PK in these cells (Supplemental Figure 5B).

In LAPC4 cells cultured in DHEA medium for 48 hours and then treated with 4 Gy IR, the peak expression of DDR proteins was measured by immunoblotting 12 hours after IR treatment. Expression levels of the DDR proteins DNA-PK, Ku80, Ku70, MRE11, and NBS were higher in *HSD3B1*- (1245C) compared with *HSD3B1*-transduced (1245A) cells (Figure 3F) after IR treatment, demonstrating induction of DDR proteins following irradiation. RNA-Seq on LAPC4 cells transduced with the *HSD3B1* (1245C) allele revealed a corresponding increase in AR-driven genes (e.g., *KLK3, KLK2*) and DDR gene expression (e.g., *BAK1, ATM, CDC25c, FKBP5, POLE*) compared with cells transduced with *HSD3B1* (1245A) allele (Figure 3G and Supplemental Figure 5C). We conducted an interaction analysis to evaluate *HSD3B1* genotype–specific DDR gene induction, independent of radiation treatment. The interaction analysis revealed a unique subset of DDR genes involved in DSB repair (e.g., *CCNA2, TOP2A, BRCA1, ATM, POLQ, BAK1, EYA1*) were upregulated in LAPC4-transduced (1245C) cells independently of the radiation treatment group (Supplemental Table 2)

### Transcriptomic analysis of DDR pathway genes in radical prostatectomy samples.

**Figure 4:**
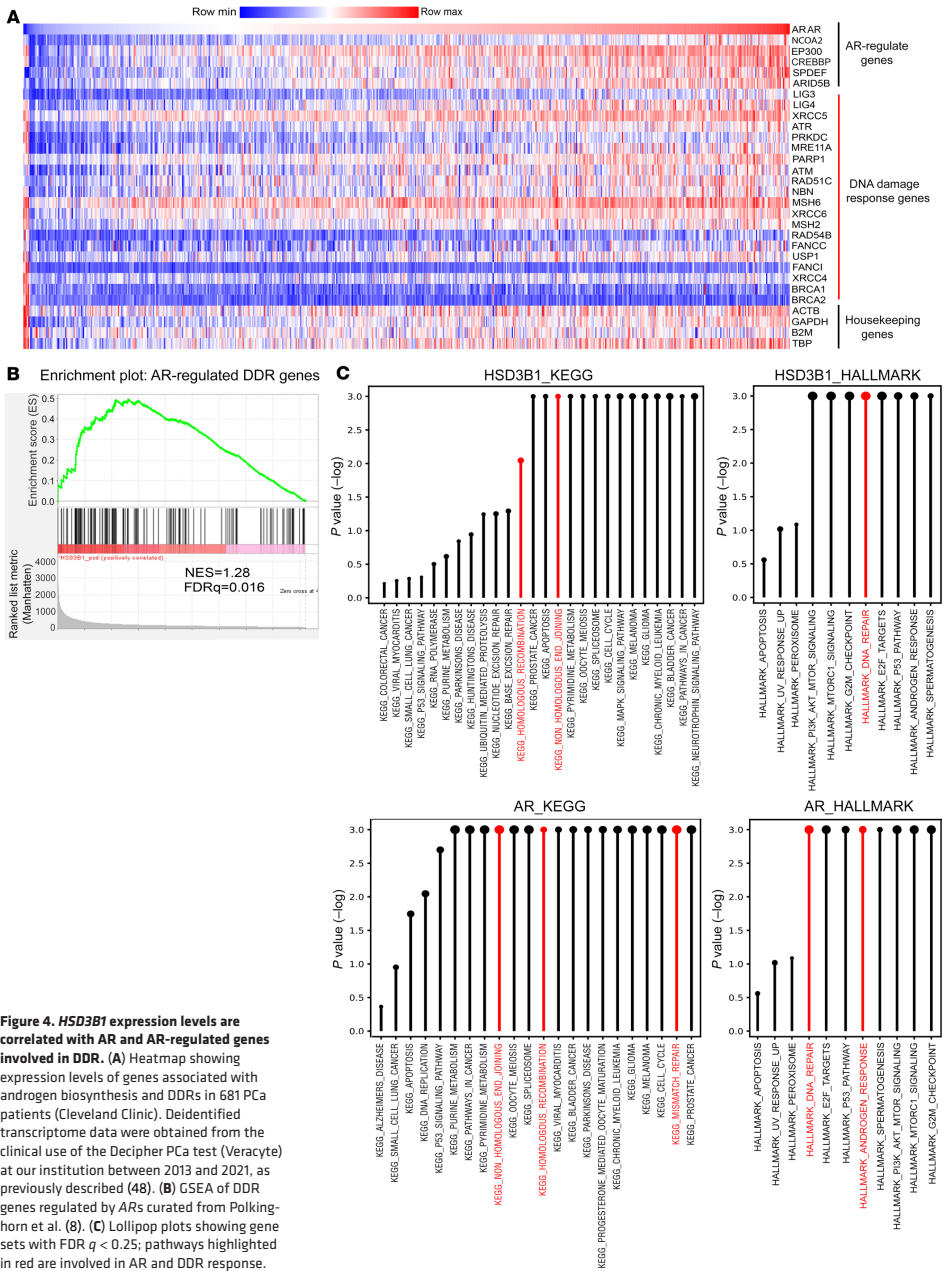
*HSD3B1* expression levels are correlated with AR and AR-regulated genes involved in DDR. (**A**) Heatmap showing expression levels of genes associated with androgen biosynthesis and DDRs in 681 PCa patients (Cleveland Clinic). Deidentified transcriptome data were obtained from the clinical use of the Decipher PCa test (Veracyte) at our institution between 2013 and 2021, as previously described (48). (**B**) GSEA of DDR genes regulated by *AR*s curated from Polkinghorn et al. (8). (**C**) Lollipop plots showing gene sets with FDR *q* < 0.25; pathways highlighted in red are involved in AR and DDR response.

Analysis of gene expression data from 681 radical prostatectomy (RP) specimens from patients treated at a single institution between 2013 and 2021 showed a correlation between *AR* expression and DDR gene expression in radiation therapy–naive patient tumors (Figure 4A). Increased *AR* expression was associated with increased expression of *XRCC5, LIG4, LIG3, PRKDC, MRE11A, MSH6, PARP1, ATM*, and other DDR genes previously associated with *AR* transcriptional transactivation (8) (Figure 4A). The maximal correlation coefficients between DDR genes and *AR* were consistent with correlation coefficients observed for other known AR-dependent genes, e.g., *NCOA2*, *EP300*, and *CREBBP* (Supplemental Table 3). The gene networks upregulated in concert with AR expression included DNA damage–sensing pathway genes as well as DDR pathway genes involved in nonhomologous end joining, homologous recombination, mismatch repair, and base-excision repair, consistent with previous reports of AR-regulated gene networks (6, 8). From our examination of the gene membership of curated DNA repair networks (Gene Ontology [GO], https://geneontology.org/; Kyoto Encyclopedia of Genes and Genomes [KEGG], https://www.genome.jp/kegg/genes.html; and Broad Institute Molecular Signatures Database [MSigDB], https://www.gsea-msigdb.org/gsea/msigdb/index.jsp), 380 genes were found to be expressed at measurable levels in our 681 RP patient transcriptome data. The heatmap shown in Figure 4A comprises a selection of DDR genes. This subset has been carefully chosen based on their membership in a recognized network of AR-regulated DDR genes (8). Gene set enrichment analysis (GSEA, https://www.gsea-msigdb.org/gsea/index.jsp) (Broad Institute) (30, 31) confirmed induction of known AR-regulated DDR gene networks in samples with high *HSD3B1* expression (Figure 4B). Additionally, selective enrichment for DNA DSB repair pathways (NHEJ and HR) with *P* < 0.05 and FDRq value of less than 25% in both *HSD3B1*-high and *AR*-high patient samples was observed (Figure 4C).

### Enza resensitizes adrenal-permissive HSD3B1-expressing PCa cells to radiation.

Enza is an orally bioavailable ARI that directly targets AR to inhibit multiple steps in the AR signaling pathway (12). We explored whether pretreatment with Enza could enhance the radiosensitivity of adrenal-permissive *HSD3B1*-expressing LNCaP cells. Cells were pretreated with 50 μM Enza (HY-70002, MedChemExpress) for 24 hours in either 50 nM DHEA, 1 nM R1881, or csFBS media. We assessed cell proliferation after 48 hours and found that Enza inhibited the growth of LNCAP shControl cells in both DHEA- (51% inhibitin, *P* < 0.005) and R1881-supplemented (63% inhibition, *P* < 0.005) media, but had no effect on cells in csFBS media (Supplemental Figure 6A).

**Figure 5:**
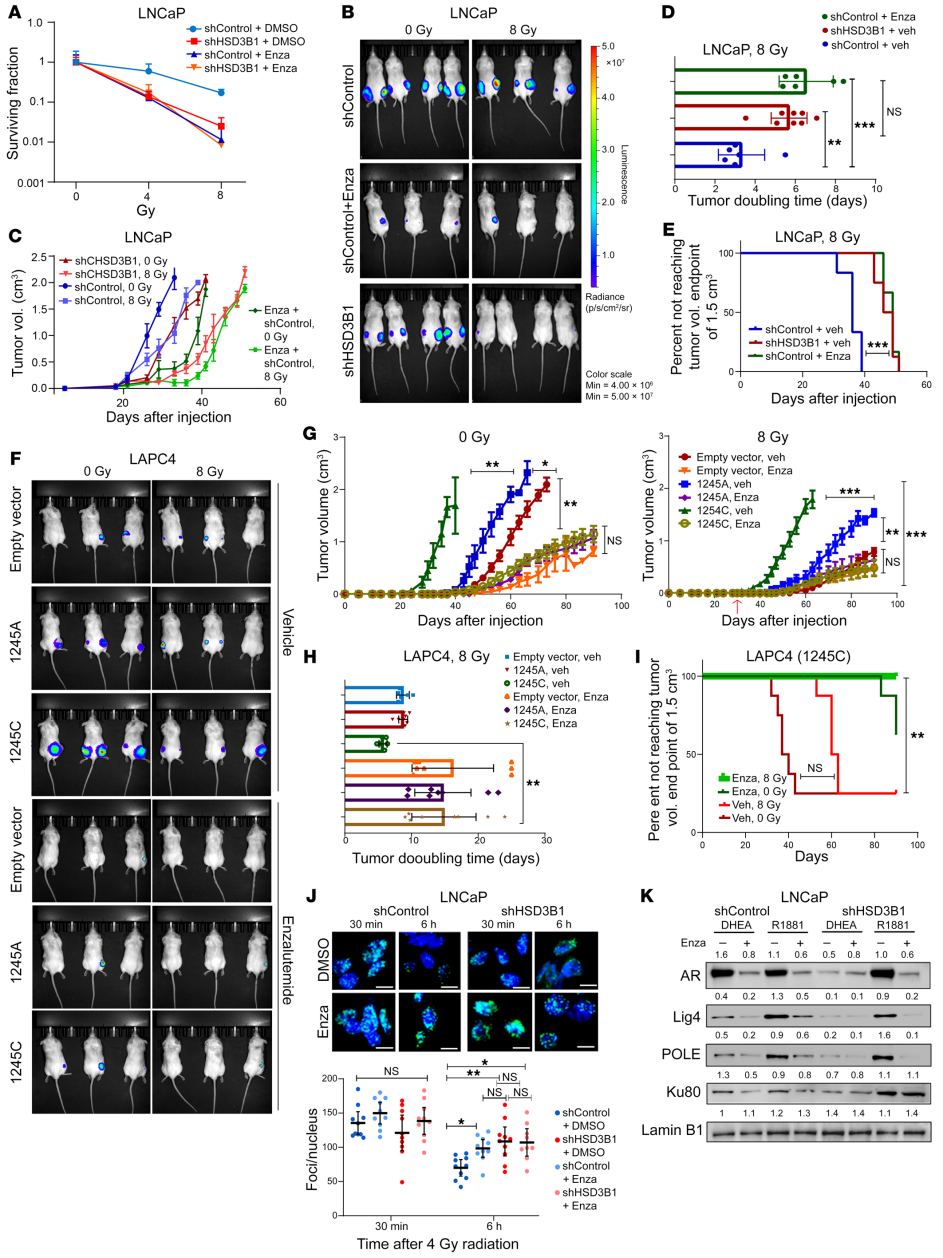
Enza pretreatment restores radiosensitivity. (**A**) Clonogenic survival of Enza-treated cells following 0, 4, and 8 Gy IR treatment. Data are represented as mean ± 95% CI of 3 technical replicates. (**B**) Representative bioluminescence images of LNCaP xenografts undergoing oral gavage with either vehicle or Enza. (**C**)Tumor volume changes over the experimental duration for shControl and shHSD3B1 xenografts after 8 Gy IR, with and without Enza (shControl; *n* = 8, shHSD3B1; *n* = 8, shControl+Enza; *n* = 6) for each 0 Gy and 8 Gy IR treatment arm. *P* values were calculated using Mann-Whitney *U* test for nonparametric data analysis. Data are represented as mean ± SEM. (**D**) Doubling time of vehicle-treated shControl and shHSD3B1 tumors compared with Enza-treated shControl LNCaP tumors. All data are represented as mean values ± 95% CI (*P* values were calculated using 1-way ANOVA with Bonferroni’s multiple-comparison test). (**E**) Kaplan-Meier curve depicting the time to meet tumor volume greater than 1.5 cc for LNCaP xenografts. *P* values were calculated using log-rank test between groups. (**F**) Representative bioluminescence images of LAPC4 xenografts undergoing oral gavage with either vehicle or Enza with or without IR (*n* = 6 for each experimental condition for each *HSD3B1* genotype). (**G**) Changes in tumor volume of LAPC4 (EV, 1245A, and 1245C) xenografts after 0 Gy (left) and 8 Gy IR (right). *P* values were calculated using Mann-Whitney *U* test for nonparametric data analysis. Data are represented as mean ± SEM. (**H**) Tumor-doubling time of LAPC4 xenografts that had undergone 8 Gy IR treatment. All data are represented as mean values ± 95% CI (*P* values were calculated using 1-way ANOVA with Bonferroni’s multiple-comparison test). (**I**) Kaplan-Meier curve depicting the time to meet tumor volume greater than 1.5 cc for LAPC4 xenografts. *P* values were calculated using log-rank test between groups. (**J**) Phospho-γ H2A.X foci formation and resolution after 4 Gy IR with Enza treatment in LNCaP cells in vitro. All data are represented as mean values ± 95% CI (*P* values were calculated using 2-tailed *t* test). (**K**) Immunoblot from LNCaP cells treated with DHEA or R1881 with or without Enza after 4 Gy IR showing Enza suppresses DDR protein expression. *P < 0.05; **P < 0.01; ***P < 0.001; ****P < 0.0001

Pretreatment with Enza not only inhibited proliferation, but also resensitized LNCaP cells to radiation. The surviving fraction of previously radioresistant shControl cells decreased (SF8_DMSO_ = 0.1703 versus SF8_Enza_ = 0.011, *P* < 0.005) following Enza treatment, approaching the SF8 levels seen in DMSO-treated shHSD3B1 cells in DHEA (SF8_DMSO_ = 0.024) (Figure 5A). Seeking to validate this finding in vivo, we randomized LNCAP xenograft animals into 3 groups: shHSD3B1 (radiosensitive group), shControl (radioresistant group), and shControl+Enza (resensitization experimental group). These groups are schematically illustrated in Supplemental Figure 6B. Xenograft tumors in Enza-treated mice exhibited significantly greater growth delay following irradiation compared with shControl xenograft tumors (Figure 5B) in vehicle-treated mice. The radiation effect on growth delay observed in shControl tumors in Enza-treated mice was comparable to that observed for radiosensitive shHSD3B1 tumors (Figure 5C and Supplemental Figure 6, C and D) with correspondingly similar mean tumor-doubling times (6.06 and 7.38 days) (Figure 5D). Additionally, the time to reach a median tumor volume greater than 1.5 cc was similar between shHSD3B1 and shControl+Enza tumors: 47 days (IQR: 44.512, 49.488 days) versus 48.33 days (IQR: 46.270, 50.397days) (*P* < 0.05) after IR (Figure 5E). In summary, pretreatment with Enza selectively restored the radiosensitivity of LNCaP shControl tumors.

Next, the effect of Enza treatment on 1245C versus 1245A allele–expressing LAPC4 xenografts was examined. LAPC4 cells (EV, 1245A, 1245C) were implanted in the flanks of NSG mice as described above (Figure 5E and Supplemental Figure 6E). Enza treatment reduced the growth rate of LAPC4 tumors and selectively restored radiosensitivity in 1245C tumors (Figure 5, E–G), as evidenced by an increase in the tumor-doubling time after radiation for Enza-treated 1245C tumors compared with vehicle-treated 1245C tumors (8.29 days versus 3.87 days) (Figure 5H). The growth delay observed for 1245C tumors after Enza treatment matched the growth kinetics of radiosensitive 1245A tumors (Supplemental Figure 6, F–H). *HSD3B1* 1245C tumor xenografts in Enza-treated animals did not reach the prespecified tumor end point of 1.5 cc after 60 days (Figure 5I). Taken together, these data support the ability of Enza to restore radiosensitivity to otherwise resistant 1245C tumors in vivo.

The DDR and kinetics of DNA DSB resolution following Enza pretreatment were also assessed. Enza treatment impaired DNA break resolution relative to vehicle-treated (DMSO) shControl LNCaP cells. The residual phosphor γ-H2AX foci counts 6 hours after 4 Gy IR in Enza-treated cells were similar to those observed in shHSD3B1 cells (Figure 5J). Immunoblots with antibodies against selected DDR genes showed that Enza pretreatment selectively suppressed DDR protein levels in DHEA media and R1881-supplemented media (Figure 5K). These findings support the hypothesis that adrenal-permissive *HSD3B1* modulates intracellular androgen pools promoting DDR-mediated radioresistance and that AR blockade with Enza abrogates this effect.

## Discussion

Germline inheritance of the adrenal-permissive *HSD3B1*(1245C) allele, which is present in approximately half of all men with advanced PCa, has been associated with more rapid progression to castrate-resistant disease (25, 32, 33). The frequency of the 1245C allele in the population shows significant variation across racial groups and can range anywhere from 53% to 4%, with a notably higher occurrence among White men (34, 35). The gene encodes a 3βHSD1 protein with a single amino acid substitution, producing a stabilized, proteasomal degradation–resistant isoform of the enzyme. 3βHSD1 increases metabolic flux through the rate-limiting step in the synthesis of potent androgens (17, 36). There is a correlation between the dosage of the C allele and progression to metastatic castrate-resistant disease in men treated with radiotherapy and ADT. Patients who are homozygous for the permissive allele (CC) experience a shorter time to metastasis compared with those with a heterozygous (AC) genotype (25). One consequence in men who inherit the 1245C allele is increased intratumoral production of T, bypassing gonadal androgen blockade. Transcriptional upregulation of AR target genes, including DDR pathway proteins, has been associated with resistance to radiation therapy (6, 8). We hypothesized that increased intracellular production of T mediated by the adrenal-permissive *HSD3B1* gene would drive more efficient DNA DSB resolution and promote resistance to radiotherapy.

In this study, we report that PCa cells expressing endogenous adrenal-permissive *HSD3B1* exhibit a radioresistant phenotype. Increased radioresistance occurred even in androgen-deprived conditions, provided cells were supplemented with the substrate for 3βHSD1 (the adrenally synthesized steroid precursor DHEA). The radiosensitivity observed in *HSD3B1*-depleted cells was fully reversed by supplementation with the synthetic AR agonist R1881. The effect persisted in vivo in castrated murine xenograft models implanted with controlled release DHEA pellets to mimic human adrenal physiology. In LAPC4 cells that harbor the adrenal-restrictive (1245A) allele, and therefore have low endogenous levels of 3βHSD1, exogenous expression of the adrenal-permissive 1245C allele resulted in increased radioresistance compared with cells expressing the adrenal-restrictive 1245A allele. This effect was confirmed in vivo, and the observation was consistent across all advanced PCa cell lines examined as well as in immortalized epithelial cells derived from the peripheral zone of a histologically normal adult human prostate (RWPE1).

Increased resistance to radiotherapy was associated with upregulation of DNA DSB repair pathway proteins (NHEJ and HR) in *HSD3B*1-expressing cells supplemented with DHEA. *HSD3B1*-expressing LNCaP cells and *HSD3B1*-expressing (1245C) LAPC4 cells demonstrated an enhanced ability to resolve lethal DNA DSBs following radiation treatment. Apart from the transcriptional upregulation of DDR genes observed here, both posttranscriptional and posttranslational modifications of DDR genes, downstream of both AR activation and irradiation, likely play a role in differential response to radiotherapy. Indeed posttranslational modifications of proteins are essential during the initial stage of DDR, as they facilitate protein-protein interactions and control protein trafficking, localization, activity, and stability (37, 38). Consistent with this assertion, accumulation of DDR proteins was observed following IR exposure hin *HSD3B1* (1245C) cells, in some cases in the absence of significant transcriptional upregulation. This finding has implications for gene expression biomarkers (39–41) that assess DDR gene activation in pretreatment samples, which may not fully capture poised states and posttranscriptional/posttranslational regulation mediating resistance in the aftermath of genotoxic treatments.

A direct association between *AR* levels and DDR gene expression was confirmed in 681 RP specimens using whole-transcriptome profiling. The association was significant and included damage response and repair genes from a range of canonical pathways. It is worth noting that there were exceptions to this rule; a subset of AR-high tumors expressed low levels of DDR genes. Our findings highlight the need for improved biomarkers of DDR capacity, perhaps combining tumor gene expression and functional repair capacity assays as well as germline and somatic genomic profiling. To this end, prospective and retrospective *HSD3B1* genotyping efforts are currently underway in patients with localized PCa receiving or having previously received combined radiotherapy and ADT.

Taken together, our results support a fundamental role for 3βHSD1 in modulating treatment response in men treated with combined androgen deprivation and radiation for PCa. In addition, we demonstrated pretreatment with a nonsteroidal selective ARI (Enza) effectively restored radiosensitivity in *HSD3B1* high-expressing cell lines in vitro and in vivo. With the exception of very high-risk and node-positive disease (42), there is currently no widely accepted standard for use of combined androgen blockade (CAB) with radiotherapy for unfavorable risk groups, for example, with a GnRH agonist and a nonsteroidal antiandrogen. Clinical trials investigating the efficacy of GnRH-directed monotherapy versus CAB with radiation therapy for unfavorable intermediate- or high-risk PCa have yielded conflicting results (42–44), leaving open the possibility that an undefined subset of patients may selectively benefit from CAB.

At present, there is no direct clinical validation of the association between 1245C HSD3B1 and radiation resistance. All clinical associations to this point have focused on the link between the adrenally permissive HSD3B1 and prognosis in advanced, metastatic disease. This may stem, in part, from historical impediments to establishing such a connection in localized hormone-sensitive disease. Confounders include variations in hormone-therapy administration, including use of ADT with or without secondary AR-directed therapy concurrently with radiation; consistent with our findings, any predictive value of 1245C would be diminished in men treated with CAB, e.g., ADT+ARSI.

An additional confounder with respect to clinical validation of biomarkers of radioresistance is the unreliable detection of local (in field) recurrence. Biochemical progression and recurrence on conventional imaging (CT/Tc99 Bonescan) have a limited sensitivity/specificity for detecting in-field radiorecurrent disease, e.g., in the prostate. The routine use of prostate-specific membrane antigen (PSMA) PET scans is likely to change this by enhancing our capacity to distinguish local and distant recurrence more reliably, creating a window of opportunity. With this in mind, prospective studies (at our center and elsewhere) will examine the role of HSD3B1 in driving radioresistance, accounting for ADT and ARSI use and tracking PSA response as well as PSMA-based patterns of recurrence. HSD3B1 genotyping is straightforward and should be considered as a correlative test in current and future clinical studies of prostate radiotherapy.

This study is the first, to our knowledge, to demonstrate a role for germline 3βHSD1 genotype in modulating radiation response in PCa. Our data suggest that patients harboring the deleterious adrenal-permissive *HSD3B1* 1245C allele may exhibit partial resistance to the radiosensitizing effects of gonadal androgen blockade alone and might benefit from CAB with radiotherapy. Prospective validation in the context of clinical trials is underway to determine whether a patient’s *HSD3B1* germline genotype might allow more precise selection of patients likely to benefit from CAB.

The growing number of highly effective PCa therapies has created a need for improved biomarkers for the rational selection of high-risk patients for earlier treatment intensification. To our knowledge, this is the first report of a germline genomic variant affecting intracellular androgen metabolism that promotes PCa cell resistance to radiotherapy under androgen-depleted conditions. This work has therapeutic implications and supports the prospective study of intensified hormonal therapy targeting extragonadal androgen biosynthesis in combination with radiotherapy for a genetically defined high-risk subset of patients with PCa.

## Methods

### Cell culture.

The human PCa cell lines LNCaP, VCaP, RWPE1, and C4-2 were purchased from ATCC and cultured according to the supplier’s guidelines. LNCaP and C4-2 cells were cultured in RPMI-1640 supplemented with 10% FBS (Gibco, Thermo Fisher Scientific) and 1% penicillin/streptomycin (pen/strep) solution (Thermo Fisher). VCaP cells were cultured in DMEM medium with 10% FBS and 1% pen/strep solution. RWPE1 was maintained in complete 1× keratinocyte medium (Gibco, Thermo Fisher Scientific) supplemented with 50 μg/mL BPE, 5 ng/mL EGF, SFM, and 1% pen/strep solution. LAPC4 cells were a gift from Charles Sawyers (Memorial Sloan-Kettering Cancer Center, New York, New York, USA) and were cultured in IMDM (Gibco, Thermo Fisher Scientific) with 10% FBS and 1% pen/strep solution. LAPC4 (also a gift from Charles Sawyers) was cultured in IMDM supplemented with 10% FBS. All cells were maintained in 1% pen/strep solution in 5% CO_2_ at 37°C except for VCaP cells, which were maintained at 8% CO_2_ in a humidified incubator at 37°C.

### Androgen deprivation and supplementation.

PCa cells were seeded in complete growth medium containing 10% FBS overnight. Cells were passaged in medium with 5% csFBS (Gibco, Thermo Fisher Scientific) for at least 48 hours to mimic androgen-deprived conditions. To simulate extragonadal androgen precursor availability, cells were grown in 5% csFBS medium supplemented with 50 nM DHEA (DHEA 700087P, Sigma-Aldrich). To mimic potent androgen-replete conditions, medium was supplemented with 1 nM R1881 (R0908, Sigma Aldrich) in 5% csFBS medium.

### Stable lentiviral shRNA and dox-induced overexpression of HSD3B1.

shRNA targeting HSD3B1(TRCN0000415869, TRC version: 2 clone ID: NM_000862.2-1120s21c1, sequence: 5′-CCGGCGTATTCACCTTCTCTTATAACTCG-3′ and 5′-AGTTATAAGAGAAGGTGAATACGTTTTTTG-3′) and a nontargeting shRNA control (SHC016) were purchased from MilliporeSigma (MISSION RNAi). Virus packaging was performed in HEK 293T cells using a second-generation lentiviral packaging system. Briefly, 0.5 μg of both psPAX2 and pMD2G plasmids and 1 μg of the target plasmid were cotransfected to produce lentiviral particles using Mirus-TransIt Lenti (MIR6655). After 48 hours, the viral supernatant was collected and filtered through 0.45 μm filters. 500 μL Aliquots were flash-frozen and stored at –80°C to avoid repeated freeze-thaw cycles. PCa cells were seeded and passaged overnight in 6-well plates (~70% confluence) in complete medium. Viral particles were mixed with 8 μg/mL polybrene (SCBT), and cells were transduced and incubated with viral particles for 72 hours. The cells were then washed and placed in an antibiotic selection medium with 1 μg/mL puromycin. After selection, the stably transduced cells were evaluated for knockdown efficiency.

To generate cells that stably expressed 3βHSD1, lentiviral plasmids with dox-inducible WT (367N) or mutant (367T) were PCR amplified by primers (forward: 5′-TCCGCGGCCGCGGAGTGATTCCTGCTA-3′; reverse: 5′-AAGACGCGTGAGCTCTAGTAGTCAAAA-3′) and subcloned into the pLVX-Tight-Puro vector (Clontech) using Not1 and Mlu1 restriction digestion. For stable dox-inducible overexpression of the adrenal-restrictive (367N) and adrenal-permissive (367T) 3β-HSD enzymes, LAPC4 and RWPE1 line cells were transduced and selected with puromycin as described above. Following selection, cells were plated in 10% Tet System Approved FBS-containing media (Clontech) supplemented with dox (1 μg/mL) for 24 hours. The cells were evaluated for the expression of the 3β-HSD1 enzyme by Western blotting.

siRNA targeting PRKDC (catalog NM_006904) was purchased from Sigma-Aldrich, and LNCaP shControl and shHSD3B1 cells were transfected with siPRKDC using X-tremeGENE siRNA Transfection Reagent (catalog 4476093001) from MilliporeSigma. Forty-eight hours after transfection, cells were evaluated for level of PRKDC knockdown and plated at clonogenic density for subsequent clonogenic survival assays.

### Phospho-γH2A.X foci quantitation.

10,000 cells per well were seeded in 24-well plates on coverslips coated with poly-l-lysine (Corning) in growth medium supplemented with steroid hormone derivatives, as previously described. After 48 hours, the plates were irradiated on a rotating platform with a single fraction of 4 Gy (Cs 137 Shepherd Irradiator) radiation. Cells were gently washed with PBS (Ca^2+^, Mg^2+^ free) at different time points and fixed with 4% paraformaldehyde for 10 minutes at room temperature. Fixed cells were washed twice with PBS and permeabilized with PBS/0.5 % Triton-X 100 for 10 minutes at room temperature, followed by washing twice with PBS-T. Anti–phospho-histone H2A.X (Ser139) antibody (clone JBW301, Millipore) diluted in PBS was added to coverslips and incubated in a humidified chamber at 4°C overnight. The coverslips containing the cells were then washed 3 times with PBS-T. Alexa Fluor 488 secondary antibody diluted (1:2,000) in PBS-T was added to the coverslips and incubated in a humidified chamber at 37°C for 20 minutes. The coverslips were washed 4 times with PBS-T before mounting on glass slides using Vectashield Mounting Medium containing DAPI (Vector Laboratories). Multiple images were captured from different fields across the coverslip and were acquired at 40×/1.25NA and 63×/1.32NA magnification using a Leica DM 6 B upright microscope equipped with a Leica 7000GT camera and LAS-X software (Leica Microsystems). Phospho-γH2A.X foci were quantified using ImageJ software (26).

### Neutral comet assay.

Cells were irradiated with 4 Gy of radiation, as mentioned above. Following irradiation, the cells were trypsinized (Trypsin, Gibco, Thermo Fisher Scientific) and resuspended in ice-cold PBS (Ca^2+^ and Mg^2+^ free) at a density of 1 × 10^5^/mL. Cells were mixed with molten low-melting agarose (Trevigen) at a ratio of 1:10 (v/v), and 50 μL of the cell suspension was immediately pipetted onto a comet slide (Trevigen) and spread evenly. Slides were placed on a level surface at 4°C in the dark for 30 minutes. The slides were immersed in a 4°C lysis solution and incubated overnight at 4°C. Excess buffer was drained from the slides and gently immersed twice in 50 mL of 1× TBE buffer for 5 minutes. The slides were placed in an electrophoresis unit and run at 16V for 40 minutes. Excess buffer was drained, and slides were immersed twice in water for 5 minutes each and then in 70% ethanol for 5 minutes. The samples were then dried at 37°C for 15 minutes. The slides were stained with SYBR Gold (Fisher Scientific, S11494) for 30 minutes and briefly rinsed with water. Slides were dried at 37°C and imaged at ×40/1.25 NA and ×63/1.32 NA using a Leica DM 6B upright microscope equipped with a Leica 7000GT camera and LAS-X software (Leica Application Suite, version 3.7.4, Leica Microsystems). The comet tail intensity and tail moment were quantified using Image J (26). Bivariate plots of comet intensity and tail moment were plotted in horseshoe plots as previously described (27). Confounders related to pseudoreplication of nested experimental units were considered; biologic replicates were performed to confirm results across independent observations from discrete experiments. Tail moments were calculated as previously described (45).

### Cell proliferation assays.

Cell confluence was measured by seeding PCa cells (20,000 cells per well) in 24-well plates at 30%–40% confluency under varying steroid hormone supplementation conditions, as described above, and proliferation was monitored using the IncuCyte Live-Cell Imaging System and software (Essen Instruments 2015A) for up to 120 hours. Proliferation kinetics were measured by seeding 5,000 cells per well (10% confluency) in 96-well plates at each time point. Cell proliferation was measured using CellTiter-GLO (Promega) according to the manufacturer’s instructions. Luminescence was measured using a BioTek Synergy plate reader (BioTek Instruments Inc.).

### Colony formation assays.

Cells were plated at clonogenic density in control and experimental media conditions and irradiated with 0, 2, 4, 6, and 8 Gy single-fraction radiation (Shepherd Mark 1 Cs-137 irradiator). The cells were left undisturbed for 14 to 20 days. Colonies were fixed and stained with 0.5% (w/v) crystal violet (MilliporeSigma, C0775) in 20% (v/v) methanol for 30 minutes, washed with water, and air dried. Three independent observers counted the colonies, with each colony consisting of more than 50 cells. The plating efficiency and surviving fractions were calculated as follows: plating efficiency (PE) = no. of colonies observed at 0 Gy/no. of cells seeded at 0 Gy; surviving fraction at X Gy (SFX) = (no. of colonies observed at X Gy/no. of cells seeded at X Gy)/PE.

### RNA isolation and qRT-PCR assays.

Total RNA was extracted using the RNeasy Mini Kit (QIAGEN, 74106), and cDNA was synthesized from 1,000 ng total RNA using SuperScript IV (Life Technologies, 18090200). qRT-PCR was performed using Fast SYBR Master Mix (Life Technologies, 4385617) on an Applied Biosystems StepOne qPCR machine. Target transcripts were quantified using the ΔΔCt method, as previously described (28), and normalized independently using both actin and GAPDH transcript levels. Primers were designed using Primer3 Input (version 0.4.0) (http://bioinfo.ut.ee/primer3-0.4.0/primer3/) and synthesized by Integrated DNA Technologies.

### Protein extraction and immunoblot analysis.

Total protein was extracted by adding RIPA buffer (Life Technologies, 89900) containing Halt protease and phosphatase inhibitors (Life Technologies, 78443) directly to cells in culture vessels after removing the growth media. The lysates were collected and centrifuged at 20,000*g* for 15 minutes to pellet debris; supernatants were collected for subsequent analysis. For nuclear and cytoplasmic protein extraction, NE-PER Nuclear and Cytoplasmic Extraction Reagents (Thermo Scientific, 78835) were used according to the manufacturer’s instructions. Proteins were quantified using BCA Protein Assay (Life Technologies, 23250). Protein concentration was normalized to 2 μg/μL using Laemmli Sample Buffer (Bio-Rad, 1610737), and samples were incubated at 95°C for 10 minutes. Samples of 15 to 20 μg were electrophoresed on polyacrylamide gels (Bio-Rad, 1610173) using the Sub-Cell GT System (Bio-Rad, 1704486) and transferred using TurboBlot (Bio-Rad, 1704150) to PVDF membranes (EMD Millipore, IPVH00010). Membranes were blocked for 1 hour in 5% (w/v) milk in Tris-buffered saline with 0.1% (v/v) Tween (TBS-T) and incubated overnight at 4°C with the primary antibody in 5% milk (antibodies listed in Supplemental Table 1). Membranes were washed for 10 minutes 3 times before being incubated for 1 hour with HRP-conjugated secondary antibody in 5% milk, and chemiluminescent signals were visualized using SuperSignal Femto (Life Technologies Corp, 34096) according to the manufacturer’s protocol.

### Cell-cycle analysis.

For cell-cycle analysis, cells were grown in 6-well plates in different androgen-containing media, as described above, washed with PBS, and fixed in 70% ethanol overnight. The cells were washed again with PBS, stained with propidium iodide following the manufacturer’s protocol (FxCycle PI/RNase Staining Solution), and analyzed by flow cytometry using a FACSCalibur machine and FlowJo software, version 10 (BD Biosciences).

### Mouse xenograft studies.

Eight- to twelve-week-old male NSG mice were injected subcutaneously with 5 million cells/flank. One week later, the mice were randomized into three 3-treatment/surgery groups: eugonadal: sham surgery, intact testis; castrated: surgical orchiectomy; and DHEA: surgical orchiectomy followed by a subcutaneously placed DHEA pellet (5 mg, 90-day sustained release). One week after surgery, each group was further randomized into 2 subgroups, radiated (8 Gy total, delivered as 2 daily fractions of 4 Gy each to the flanks) or sham treated (unirradiated). After radiation, tumor growth was measured 3 times a week with calipers and imaged once a week with IVIS (spectrum) in vivo bioluminescent imaging. The animals were monitored until the tumors reached a maximum volume of 2.5 cm^3^. For LAPC4 xenografts, male NSG mice were subcutaneously injected with 3 million cells/flank; 1 week later, all the mice underwent surgical orchiectomy followed by subcutaneous DHEA pellet insertion. Each group was randomized into 2 subgroups, one that received a cumulative dose of 8 Gy as above and another sham irradiated (0 Gy). Tumor growth was measured as described above. Dox (2 mg/mL) was added to the drinking water along with 1% sucrose. The drinking water was replaced weekly.

For the Enza-treated LNCaP and LAPC4 xenografts, 5 million cells/flank and 6 million cells/flank, respectively, were injected in male NSG mice. The animals underwent orchiectomy followed by DHEA pellet implantation; they were also randomized into a radiated subgroup (8 Gy) and a sham-treated group (0 Gy). Each group was further randomized into subgroups that received vehicle treatment and another group where animals were gavaged with Enza at a dose of 0.42 mg/mice/d via 20-gauge flexible plastic tubing oral gavage needles. The tumor-doubling time was assessed using the following formula (29): doubling time (DT) = log_2_ (T2 – T1)/log_2_ (V2log_2_ V1), where T2 = final time point, T1 = initial time point, V1 = initial tumor volume, and V2 = final tumor volume. The tumor measurements were staggered, as the time to reach experimental tumor volume end point differed among different experimental conditions.

### Whole-transcriptome RNA-Seq.

Total RNA was purified using a NucleoSpin RNA Kit (Takara Bio USA Inc., catalog 740955), according to the manufacturer’s instructions. RNA quality was validated by the RNA integrity number (RIN >9) calculated using the Agilent 2100 Bioanalyzer. mRNA was enriched using oligo(dT) beads and then fragmented randomly in fragmentation buffer. First-strand cDNA synthesis was performed using random hexamer primers, after which a custom second-strand synthesis buffer (Illumina, dNTPs, RNase H, and DNA polymerase I) was added to initiate second-strand synthesis. After a series of terminal repair, ligation, and sequencing adapter ligation steps, the double-stranded cDNA library was synthesized by size selection and PCR enrichment. The qualified library was loaded onto the flow cell of the Illumina sequencer after pooling indexed samples, and more than 30 million reads were acquired for each sample. The quality of the RNA-Seq raw reads was verified using prealignment QA/QC. Raw reads were mapped to the human genome, hg38, using STAR-2.7.3a alignment. Differential expression and GSEA were performed using DEseq2 and GSEA (Broad Institute) (30). Gene-specific analysis was performed using Partek Flow software, version 10.0.

### Transcriptome analysis of PCa patient samples.

Prospectively collected data from a cohort of RP patients who underwent Decipher testing from 2009 to 2020 were reviewed (31). Whole transcriptome array profiling was performed on the highest GG index lesion within the RP specimen (Decipher, Veracyte Inc.) (32). A risk score based on a clinically validated 22-gene signature was determined for each RP specimen (33–35). Normalized whole-transcriptome microarray data were obtained (Decipher, Veracyte Inc.) and analyzed using GSEA and Morpheus (Broad RRID:SCR_017386).

### Statistics.

All experimental results represent observations from at least 3 biological replicates unless otherwise indicated; all data are represented as mean ± 95% CI, and the number of replicates for each experiment is indicated in the legends. Issues related to pseudoreplication of nested experimental units were considered where appropriate (e.g., comet assay, phospho-γH2A.X foci); biologic replicates were performed to confirm results across independent observations from discrete experiments (46). All statistical analyses were performed using GraphPad Prism. Statistical significance was calculated by unpaired, 2-tailed *t* test, and *P* values of less than 0.05 were considered significant. Multiple testing corrections, if necessary, were performed using Bonferroni’s correction. Nonparametric data (e.g., volume) were compared using the Mann-Whitney *U* test. Progression-free survival was determined by Kaplan-Meier analysis followed by log-rank testing to ascertain between-group differences.

### Study approval.

All mouse studies were performed under a protocol approved by the IACUC of the Cleveland Clinic Lerner Research Institute. All human tissues were obtained at the Cleveland Clinic under IRB-approved protocols (CCF IRB 18-677).

### Data availability.

All data needed to evaluate the conclusions in the paper are available in the main text or the supplemental material. Values for all data points in graphs are reported in the Supporting Data Values file.

## Author contributions

S Ganguly performed experiments, curated data, performed formal analysis, performed data validation, designed experimental methodology, wrote the original draft of the manuscript, and reviewed and edited the manuscript. ZL performed gene expression data analysis and reviewed and edited the manuscript. AM performed experiments. AH assisted with experimental methods and reviewed and edited the manuscript. JI performed data analysis. MP and MB assisted with murine experiments. TDS, ED, KLS, JC, CJW, S Gupta, CAR, RDT, and AAC provided project guidance and reviewed and edited the manuscript. EAK conceived the project and provided resources. NS conceived the project, provided resources, supervised the project, and wrote and edited the manuscript. OYM conceived the project, provided resources, provided formal analysis, supervised the project, wrote the original draft of the manuscript, performed project administration, and reviewed and edited the manuscript. All authors approved the final manuscript.

## Supplementary Material

Supplemental data

Supporting data values

## Version 1 11/15/2023

Electronic publication
