# Supplementary material for: Intratumoral androgen biosynthesis associated with 3β-hydroxysteroid dehydrogenase 1 promotes resistance to radiotherapy in prostate cancer
Source: J Clin Invest. 2023 Nov 15;133(22):e165718. doi: 10.1172/JCI165718 (PMC10645386; doi:10.1172/JCI165718)
Supplement: Supplemental data [file jci-133-165718-s111.pdf]

Supplementary Figures and Figure legends

Supplementary Figure 1

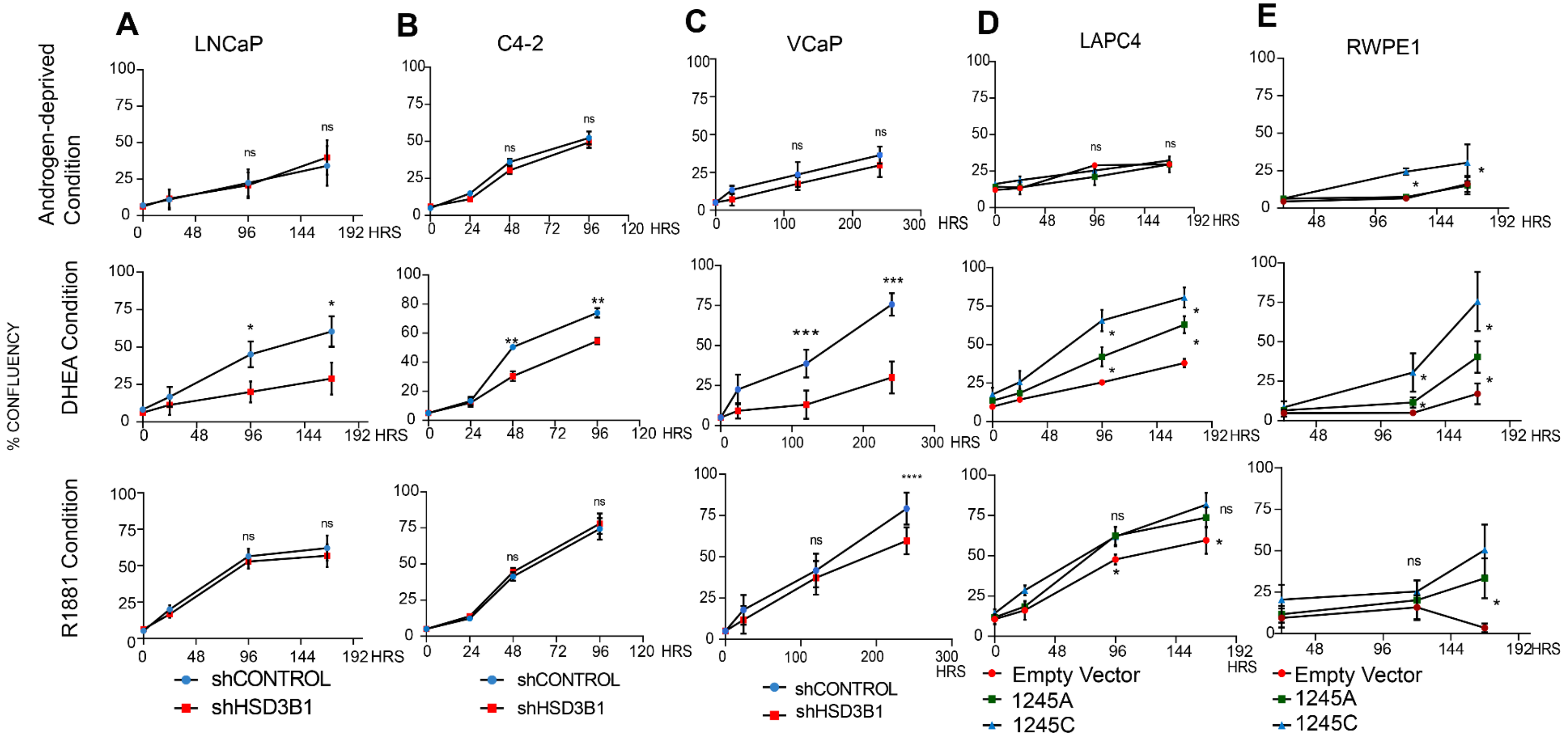

Figure S1: Adrenal-permissive HSD3B1 (1245C) expression promotes in vitro growth of prostate cancer cells. (A) Proliferation of LNCaP cells stably expressing shRNA targeting *HSD3B1* (shHSD3B1) or non-silencing shRNA (shCONTROL) cultured in charcoal-stripped FBS (csFBS) medium containing ethanol (top panel) (Androgen-deprived condition), 50nM DHEA (middle panel), or 1nM R1881 (bottom panel). (B) C4-2 and (C) VCaP cells. All data are presented as mean values ± 95% CI from triplicates of two independent experiments. * $P < 0.01$, n.s. not significant (unpaired t-test). (D) LAPC4 and (E) RWPE1 stably expressing 3βHSD1 (1245A) or 3βHSD1 (1245C). Viability was measured at the indicated times using CellTiter-Glo assay.. All data are presented as mean

values ± 95% CI from triplicates of two independent experiments for (D) and quadruplicates of two independent experiments for (E) (Multiple t-test 1 per row) * P < 0.01, n.s. not significant.

Supplementary Figure 2

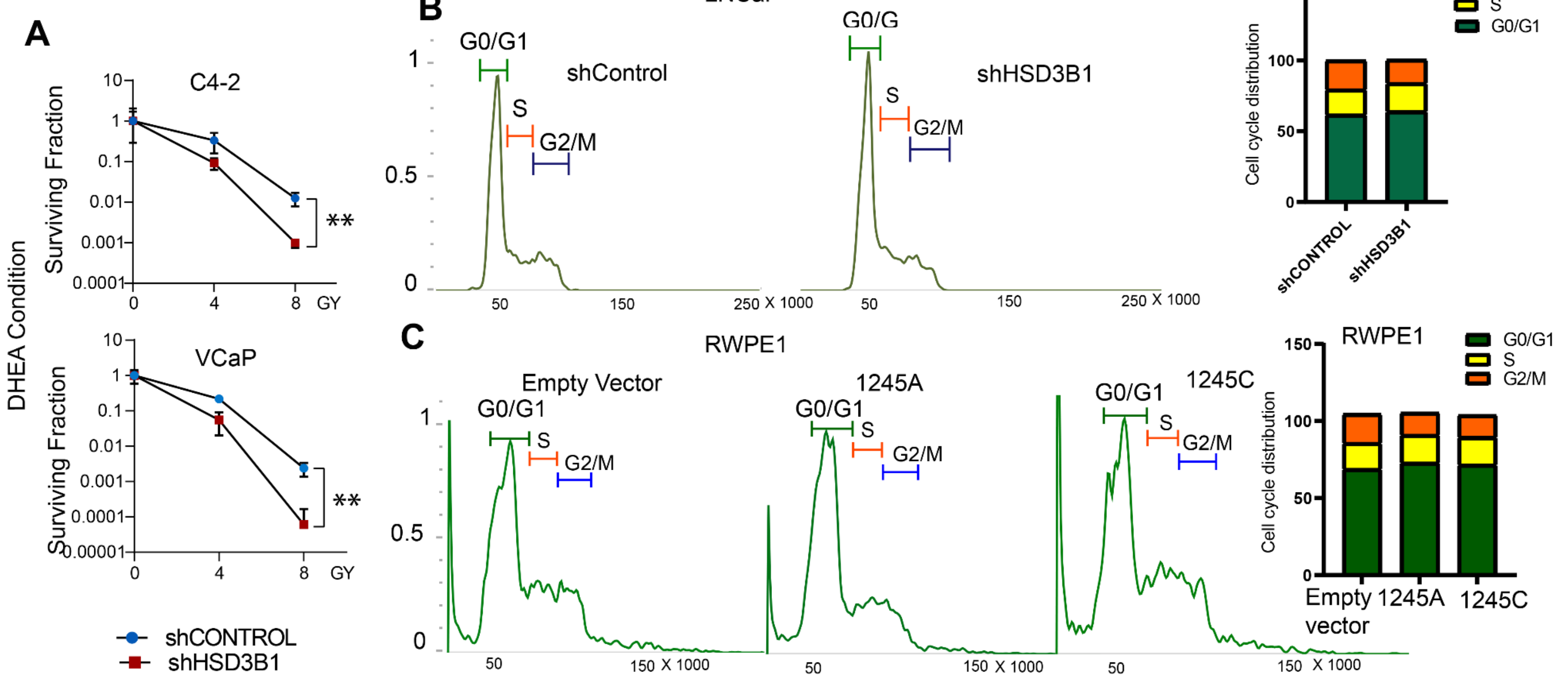

Figure S2: Knockdown of 3βHSD1 does not alter cell cycle progression in prostate cancer cells. (A) Colony formation assay of C42 (top panel) and VCaP cells (bottom panel) expressing shHSD3B1 or shCONTROL. Cells were treated with, 4, , or 8 Gy radiation and cultured for 14 days in 50nM DHEA and colonies were stained with Crystal Violet. All data are presented as mean values ±95% CI from triplicates in each two independent experiments. * $P < 0.05$, ** $P < 0.01$, **** $P < 0.0001$, n.s. not significant (unpaired two tailed t-test). FACS cell cycle analysis of LNCaP cells expressing shHSD3B1 or shCONTROL(B) and RWPE1 cells expressing *HSD3B1* 1245A, 1245C or empty vector (C) following treatment with 50nM DHEA for 48hrs. The percentage distribution of cells in each phase of the cell cycle is shown.

Supplementary Figure 3

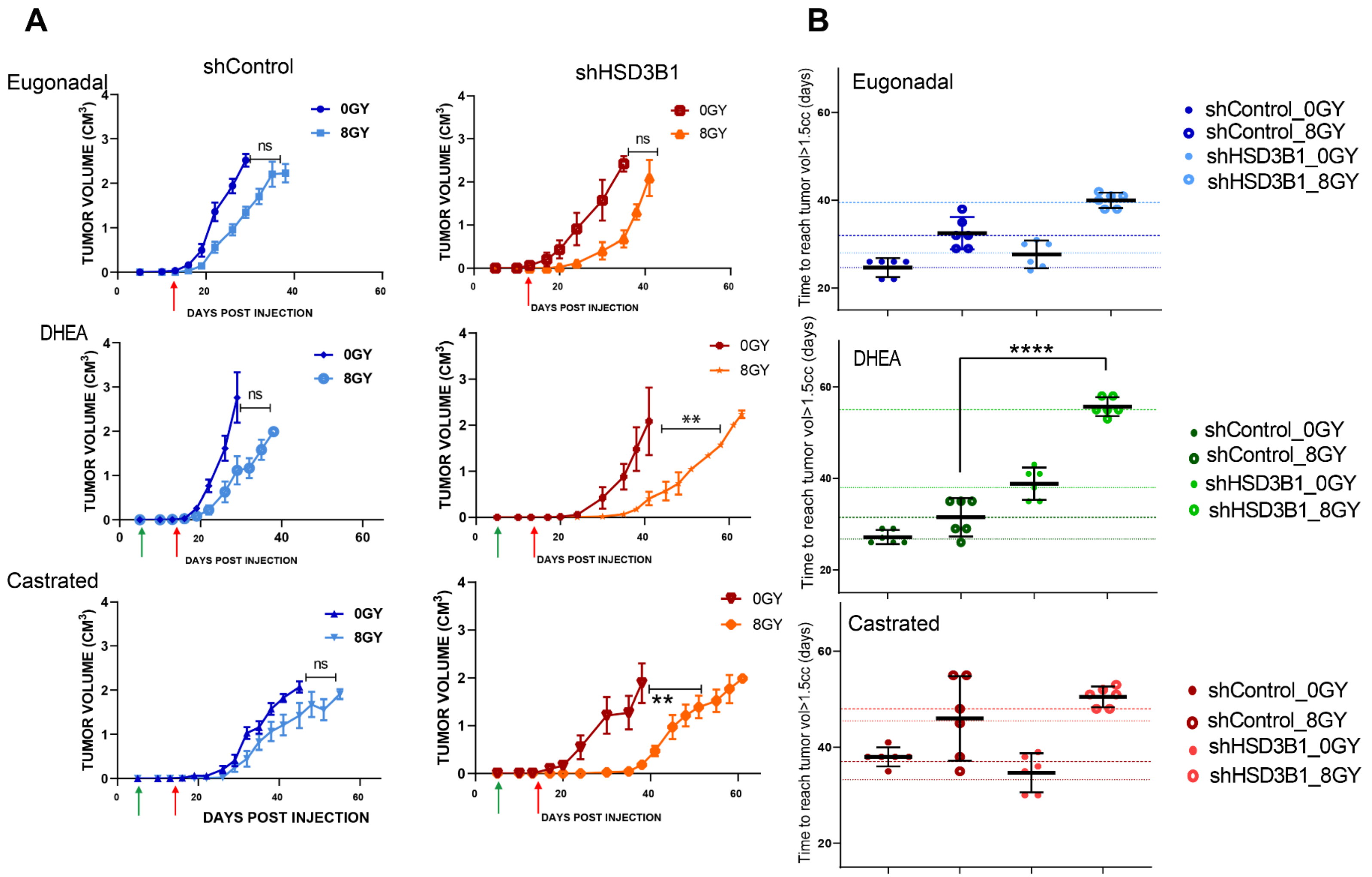

Figure S3: Loss of 3βHSD1 expression sensitizes LNcaP xenografts tumors to radiation treatment. (A) Tumor growth of shControl (left panels, n=6) and shHSD3B1 (right panels, n=6) LNCaP xenografts after sham irradiation or 8 Gy radiation in eugonadal mice,

castrated, and DHEA-supplemented mice. Red arrow: time of irradiation, Green arrow: time of surgery. Data are represented as mean ± SEM. P-values were calculated using Mann-Whitney test for non-parametric data analysis (B) Average number of days after irradiation for shcontrol and shHSD3B1 LNCaP tumors grown in eugonadal or castrated mice (alone or DHEA pellet implant) to reach and endpoint size of 1.5cm3. Data is represented as mean ± 95%CI (1-way ANOVA with Bonferroni's multiple comparison test). ** $P < 0.001$, ****$P<.0001$, n.s. not significant.

Supplementary Figure 4

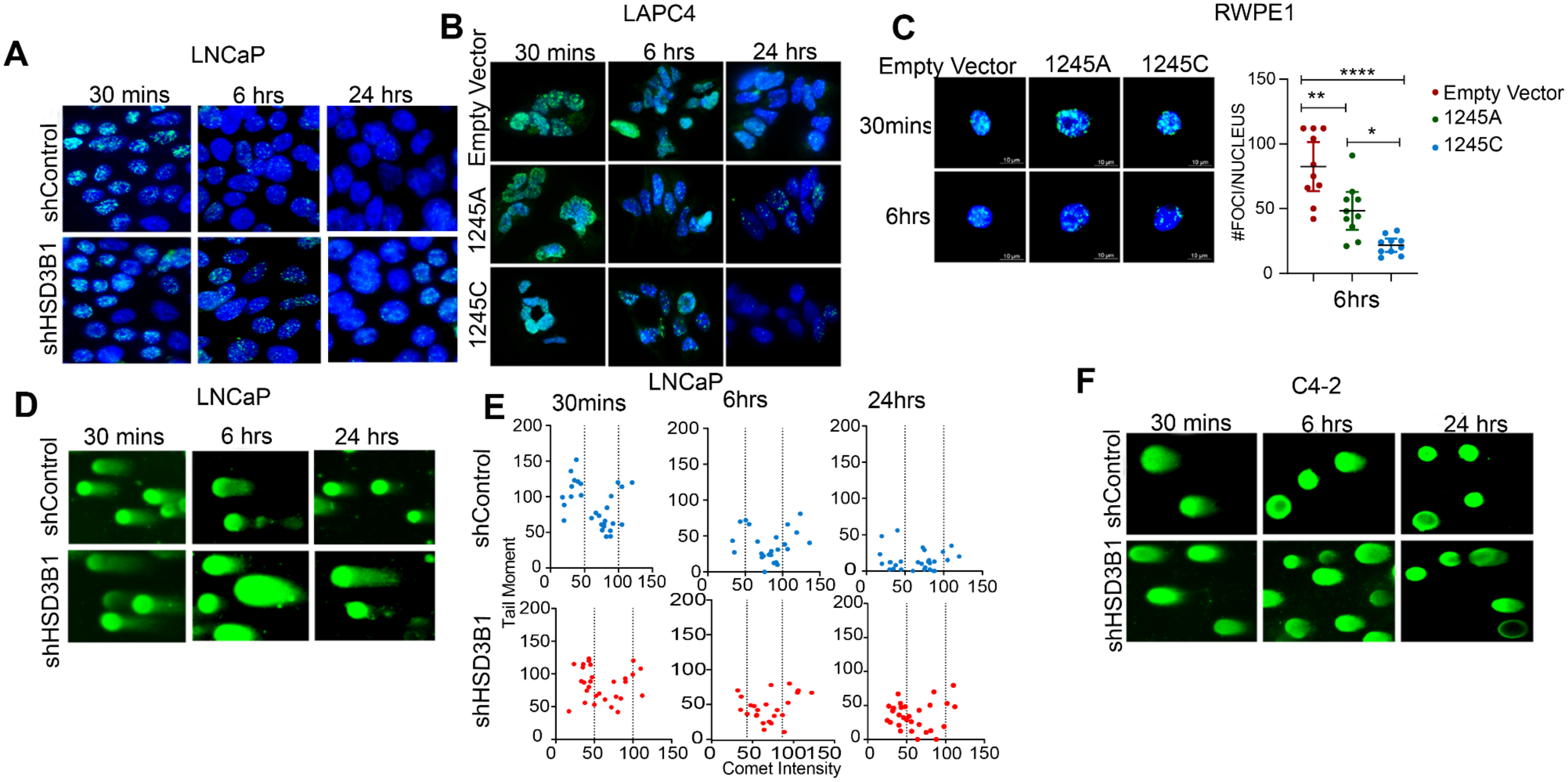

Figure S4: Effects of 3βHSD1 expression in prostate cancer cells following 4Gy radiation treatment. (A) Representative immunofluorescence images of γH2AX foci after irradiation in shCONTROL or shHSD3B1 LNCaP cells pre-treated with 50nM DHEA. (B) γH2AX foci formation in LAPC4 cells expressing Empty Vector (EV), 1245A, and 1245C *HSD3B1* alleles pre-treated with DHEA followed by irradiation (4Gy). (C) γH2AX foci formation in RWPE1 cells expressing EV, 1245A, and 1245C *HSD3B1* allele pre-treated with DHEA followed by irradiation (4Gy). All data are presented as mean values ± 95% CI (2-way ANOVA with Bonferroni's multiple comparison test). (D) Neutral COMET tail formation images of LNCaP shCONTROL and shHSD3B1 cells pre-treated with DHEA

followed by irradiation (4Gy) and (E) Bi-variate Horse-shoe plots of the same LNCaP cells after neutral COMET electrophoresis. (F) Neutral COMET tail formation images of C4-2 cells pre-treated with DHEA followed by irradiation (4Gy). All data are presented as mean values ± 95% CI. (Two-tailed t-test) *** P< 0.001, **** P< 0.0001, n.s. not significant.

Supplementary Figure 5

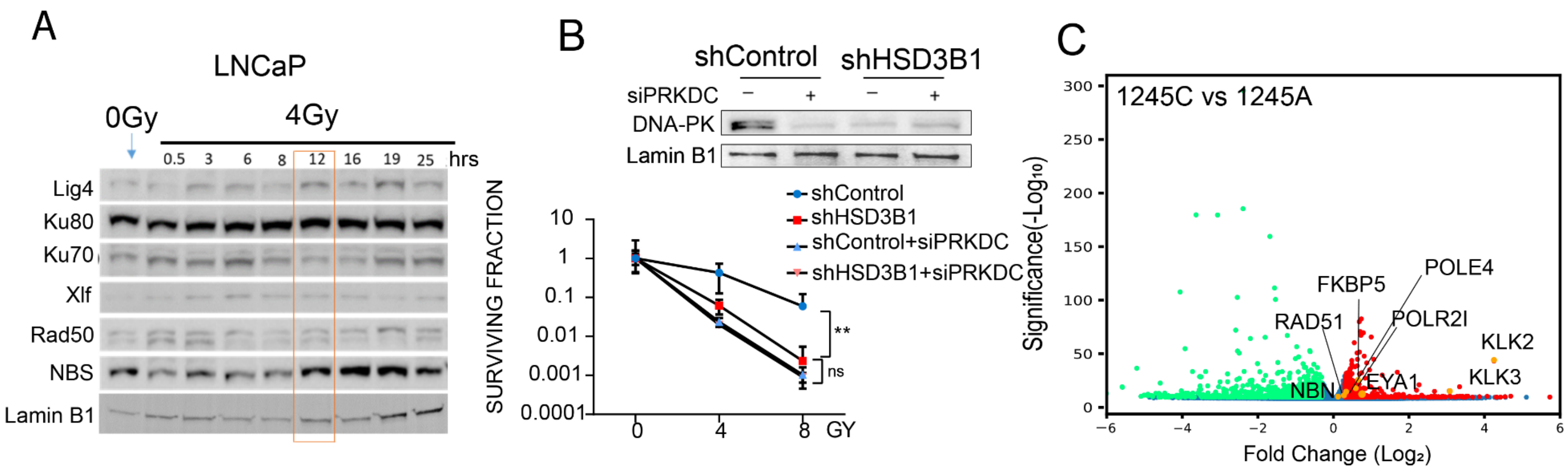

Figure S5: Kinetics of DNA Damage Response Proteins in prostate cancer cells that express mutant 3βHSD1. Western blot analysis showing the kinetics of DDR response proteins in (A) shCONTROL LNCaP cells (endogenously mutant HSD3B1) after being cultured with 1nM R1881 followed by 4Gy radiation treatment. (B) Western blot showing siRNA mediated knock-down of PRKDC in LNCaP cells cultured with 50nM DHEA (top). Colony formation assay of LNCaP cells ± siPRKDC with 0, 4, or 8 Gy radiation and cultured for 14 days in 50nM DHEA and colonies were stained with Crystal Violet. All data are presented as mean values ± 95% CI from triplicates of two independent experiments (P-value determined via unpaired t-test). (C) Volcano plots depicting differentially expressed genes in

HSD3B1 (1245C) LAPC4 compared to *HSD3B1* (1245A) LAPC4 cells. Key DDR genes are highlighted in yellow. P-values were calculated using two tailed t-test.

Supplementary Figure 6

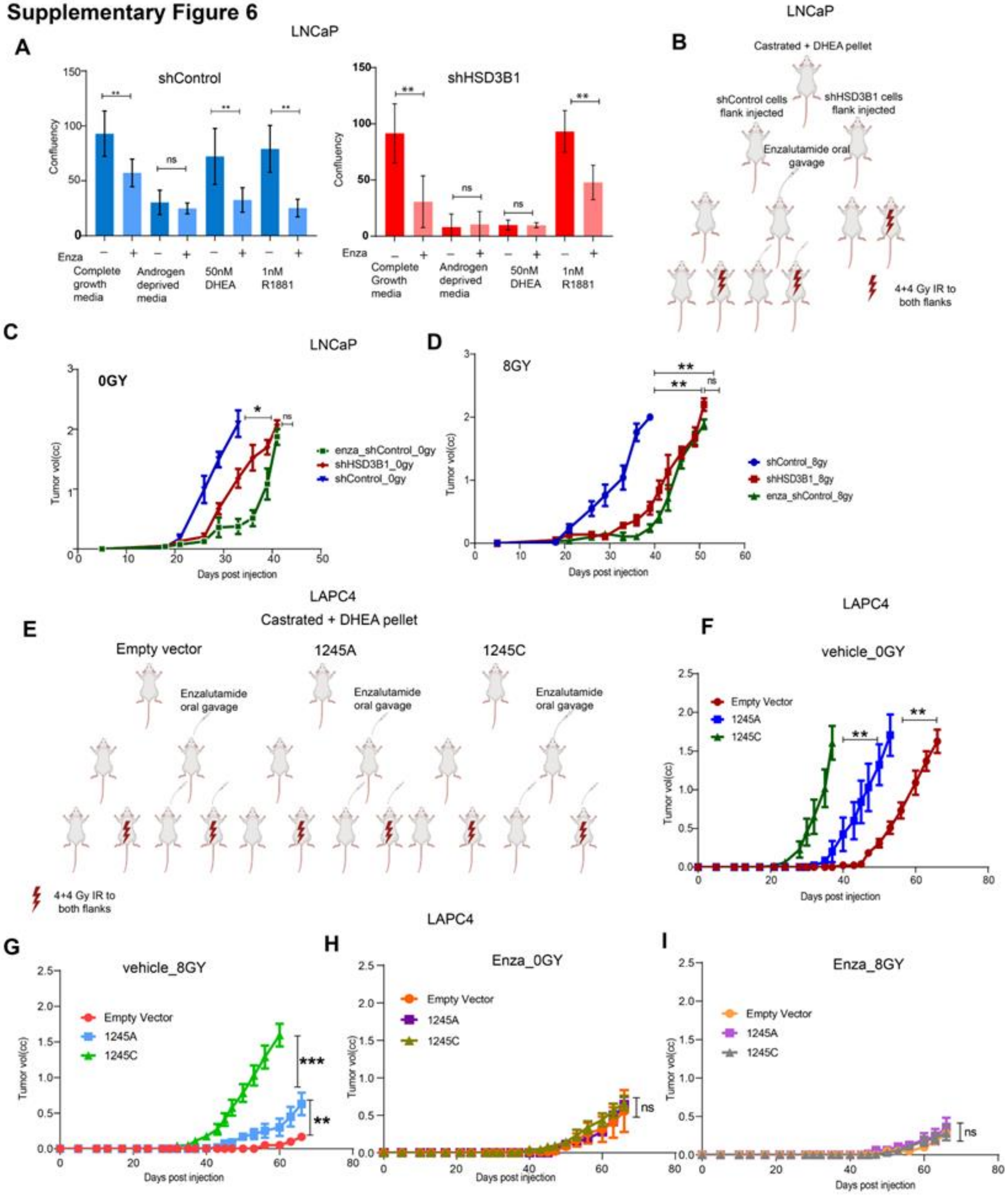

Figure S6: Enzalutamide treatment resensitizes HSD3B1 harboring cells to radiation. (A) shControl and shHSD3B1 LNCaP cells were cultured in media containing 10% FBS (complete media), csFBS (androgen-deprived media), or csFBS media containing 50nM DHEA or 1nM R1881. Proliferation was measured in cells treated with DMSO (vehicle) or 50µM Enzalutamide (Enza) after 48 hours using CellTiter-Glo assay. All data are presented as mean values ± 95% CI from triplicates of two independent experiments. * $P < 0.01$, n.s. not significant (unpaired t-test). (B) Schematic of experimental layout of LNCaP xenografts treated with Enza. (C) Tumor growth of shControl and shHSD3B1 LNCaP xenografts after treatment with vehicle or enzalutamide followed by 0Gy or(D) 8 Gy IR. Data are represented as mean ± SEM. P-values were calculated using Mann-Whitney test for non-parametric data analysis. (E) Schematic of experimental layout of LAPC4 xenografts treated with Enza. Tumor volume of LAPC4 cells harboring Empty Vector, 1245A and 1245C allele xenografts; vehicle treated-0Gy (F), vehicle-treated-8Gy (G), Enza-treated-0Gy (H), Enza-treated-8Gy (I). Data are represented as mean ± SEM. Pp-values were calculated using Mann-Whitney test for non-parametric data analysis.* $P<0.05$, ** $P<0.01$, n.s. not significant.

Supplementary table 1

**Antibody**	**Company**	**Catalogue #**
Anti-HSD3B1	Abcam	55268
Anti-Lig4	Proteintech	12695
Anti-Ku80	Proteintech	16389
Anti-Ku70	Santacruz	17789
Anti-NBS	Cell signaling technologies	3001t
Anti-POL E	Thermo Fisher	pa578113
Anti-MSH6	Proteintech	18120
Anti-MRE11	Cell signaling technologies	4847t
Anti-Rad50	Cell signaling technologies	3427t
Anti-XLF	Cell signaling technologies	2854t
Anti-lamin B1	Proteintech	12987-1-AP
Anti-ɣ H2ax	Millipore	05-636

Supplementary Table 1: List of antibodies used in this research project.

Supplementary table 2

Gene name	P-value (0GY,1245 C vs 0GY,EV)	Log2(Ratio) (0GY,1245 C vs 0GY,EV)	P-value (4GY,1245 C vs 4GY,EV)	Log2(Ratio) (4GY,1245 C vs 4GY,EV)	P-value (4GY,1245 C vs 4GY,EV)vs (0GY,1245	Log2(Ratio) (4GY,1245 C vs 4GY,EV)vs (0GY,1245

					C vs 0GY,EV)	C vs 0GY,EV)
ABL1	0.051443	0.144403	0.166316	0.104018	0.616139	-0.06036
ABRAXAS 1	0.948379	0.014258	0.255137	-0.26773	0.442228	-0.30227
ACD	0.586728	-0.05759	2.87E-15	0.836218	1.94E-06	0.87343
ACTB	0.078097	-0.10652	0.018397	-0.14283	0.766265	-0.05557
ACTL6A	0.808515	0.018835	0.00041	0.291688	0.060747	0.253189
ACTR5	0.652425	0.062766	0.800084	-0.03699	0.584052	-0.11974
ACTR8	0.952149	-0.00568	6.44E-06	-0.46436	0.00442	-0.47877
ADPRS	0.15196	-0.15016	0.011679	-0.26491	0.45009	-0.13495
ALKBH1	0.796275	0.045883	0.010311	0.483113	0.188368	0.417214
ALKBH2	0.251354	-0.12859	0.001606	-0.37814	0.143348	-0.26951
ALKBH3	0.674731	-0.06135	0.015083	-0.39027	0.170556	-0.34844
ALKBH5	0.117105	-0.11083	1.91E-14	-0.54955	0.000282	-0.45821
APAF1	0.000109	0.367807	2E-09	0.61027	0.175511	0.222454
APBB1	0.03758	-0.3035	3.31E-24	-1.61832	7.84E-08	-1.33272
APEX1	0.141817	-0.08347	3.63E-08	-0.32239	0.023148	-0.25885
APEX2	0.778296	0.032201	2.26E-05	-0.49699	0.002501	-0.54853
APTX	0.001966	-0.24717	9.82E-58	-1.40188	1.06E-17	-1.173
AQR	0.945131	0.005887	0.252003	0.105034	0.592099	0.079632
ASCC1	0.940611	0.009568	0.419643	-0.10881	0.52009	-0.13712
ASCC2	0.507151	0.055576	0.461765	-0.06336	0.335652	-0.13821
ASCC3	0.000358	0.270599	8.76E-05	0.310198	0.872201	0.020051
ATF2	0.087819	0.210893	0.011354	-0.33128	0.005451	-0.56148
ATM	9.93E-06	0.386157	0.014225	0.222985	0.205735	-0.18239
ATP23	0.783483	-0.08073	0.7695	0.094972	0.760794	0.154848
ATR	0.207578	0.123105	0.019587	-0.24396	0.028258	-0.38728
ATRIP	0.263346	-0.15176	0.000553	-0.47544	0.1167	-0.34468
ATRX	0.069046	0.17413	0.02173	-0.23504	0.006448	-0.42842
BABAM1	0.421475	-0.06655	0.218365	0.105254	0.279801	0.15144
BABAM2	0.207594	-0.14179	5.76E-07	-0.61232	0.008753	-0.49052
BAK1	0.005251	0.82366	1.25E-24	4.387998	2.65E-10	3.528166
BAP1	0.071095	-0.11242	7.49E-06	-0.28619	0.077109	-0.19391
BARD1	0.097834	0.134655	0.064006	-0.15832	0.014529	-0.31311
BAX	0.89524	-0.01796	0.15248	0.202897	0.35428	0.200408
BAZ1B	0.707732	-0.02139	0.003843	-0.17637	0.103662	-0.17434
BID	0.000826	-0.3165	0.77081	-0.02847	0.102583	0.267588
BLM	0.032087	0.316763	0.050089	0.319205	0.942208	-0.01829
BRCA1	7.25E-06	0.348596	0.072214	-0.14574	2.98E-05	-0.51423
BRCA2	0.012464	0.273405	0.003094	-0.35457	0.000292	-0.64742
BRCC3	0.615834	-0.0645	0.597099	-0.07462	0.889926	-0.02899
BRIP1	0.000023	0.400039	0.100772	-0.16673	0.000479	-0.58617

CASP3	0.332818	-0.07773	0.000008	0.392963	0.00091	0.450685
CASP9	0.250496	-0.18823	0.037599	-0.3411	0.534949	-0.17305
CCNA2	1.51E-11	0.596609	1.17E-08	0.518058	0.469394	-0.09777
CCNH	0.548333	0.087567	0.017981	-0.38425	0.052719	-0.4885
CDC25C	0.003998	0.353454	2.1E-14	0.993922	0.002904	0.620325
CDK2	0.035249	0.15635	0.000483	-0.27065	0.000188	-0.44677
CDK5	0.400432	-0.19032	0.350391	0.215202	0.33029	0.386967
CDK7	0.580908	-0.08992	0.287545	0.182878	0.35671	0.254141
CDKN1A	0.312692	0.175437	5.16E-13	0.986212	0.000988	0.789992
CDKN2A	1.78E-06	-0.81367	4.38E-18	-1.47946	0.023499	-0.68539
CENPS	0.860237	0.022444	0.908224	0.016471	0.905387	-0.02627
CENPX	0.003	-0.18646	0.09483	-0.10779	0.593581	0.058909
CETN2	0.00697	-0.27488	0.868237	-0.01881	0.201861	0.237421
CETN3	0.983465	-0.00274	0.5406	-0.09357	0.623887	-0.10988
CHAF1A	0.077629	0.134521	0.193128	-0.10348	0.029223	-0.25787
CHAF1B	0.611867	0.045077	0.649478	-0.04248	0.468435	-0.10771
CHD1L	0.946517	0.004878	0.003287	-0.23321	0.041956	-0.25811
CHEK1	0.028501	0.216138	0.074928	-0.19044	0.015556	-0.42563
CHEK2	0.611682	-0.06364	0.68951	-0.05318	0.964286	-0.00954
CIB1	0.268302	-0.08333	0.090999	0.131155	0.107212	0.194696
CLSPN	0.015882	0.204688	5.29E-07	-0.46621	1.85E-06	-0.69015
COPS2	0.936623	-0.00643	0.152004	0.12721	0.411219	0.114538
COPS3	0.710311	-0.02684	0.046442	-0.1527	0.249992	-0.14496
COPS4	0.943065	-0.00666	0.037913	0.212707	0.233504	0.198922
COPS5	0.865499	0.012917	0.229619	0.09769	0.614715	0.065488
COPS6	0.008043	-0.19183	0.189071	-0.10054	0.573584	0.071592
COPS7A	0.049751	-0.1952	0.005145	-0.29001	0.517822	-0.11396
COPS7B	0.698061	0.042295	0.187148	-0.14858	0.252463	-0.21052
COPS8	0.420094	-0.06788	0.589376	-0.04857	0.99797	-0.00037
CRY1	0.053847	-0.14352	1.23E-08	0.433138	2.76E-06	0.556695
CRY2	0.398445	-0.08924	0.330031	0.101055	0.346953	0.169365
CSNK1D	0.052118	-0.10526	0.03817	-0.11289	0.850787	-0.02747
CSNK1E	0.05502	-0.11714	0.654062	-0.02742	0.543282	0.070052
CUL4A	0.073536	0.418129	0.370314	-0.22315	0.115011	-0.65644
CUL4B	0.891298	-0.01092	0.139918	-0.1259	0.288021	-0.13452
DCLRE1A	0.000363	0.370186	2.9E-10	-0.71135	2.89E-09	-1.10169
DCLRE1B	0.149753	-0.16334	0.529735	-0.07755	0.730705	0.065523
DCLRE1C	0.66136	0.055137	0.314889	0.137161	0.764901	0.062448
DDB1	0.290463	-0.05094	7.29E-16	-0.39845	0.013408	-0.36746
DDB2	0.762274	0.041786	0.435867	0.114688	0.813698	0.054335
DMC1	0.630334	-1.14039	0.73214	0.63326	#N/A	#N/A
DNA2	0.000901	0.333896	0.760044	0.032836	0.066726	-0.32074

DTL	0.640174	0.038895	3.48E-06	-0.42285	0.000854	-0.48136
DUT	0.560677	0.047525	0.004177	-0.23403	0.023761	-0.30143
E2F1	0.05457	-0.13829	0.434229	-0.05699	0.587477	0.061391
ELL	0.023243	-0.22602	0.081956	0.174694	0.023014	0.379784
EME1	0.293916	0.14595	0.898193	0.01844	0.532221	-0.14562
EME2	0.183112	-0.12387	3.38E-10	-0.56376	0.000951	-0.45959
ENDOV	0.436316	-0.08726	0.080135	0.203643	0.139496	0.271276
EP300	0.465554	0.053671	0.090429	0.126852	0.658555	0.053514
ERCC1	0.882635	-0.01366	0.010095	-0.24908	0.111394	-0.2553
ERCC2	0.5461	-0.04873	0.007398	-0.2181	0.157481	-0.19047
ERCC3	0.051346	-0.14935	0.279308	-0.08697	0.729694	0.042852
ERCC4	0.800794	-0.03123	3.12E-05	-0.57083	0.007542	-0.55904
ERCC5	0.393267	0.143115	0.681715	0.073235	0.768576	-0.08821
ERCC6	0.217423	-0.13058	0.323043	0.113192	0.233514	0.223976
ERCC8	0.788488	0.043127	0.94435	-0.01214	0.780395	-0.07558
EXO1	0.327541	0.082411	1.15E-14	-0.68954	6.16E-08	-0.79134
EYA1	5.18E-06	0.895741	3.54E-10	-1.58487	1.52E-10	-2.50077
EYA2	0.025687	-0.3986	6.83E-24	2.158831	7.36E-14	2.536918
EYA3	0.019979	0.206018	0.741108	-0.03035	0.101448	-0.25638
FAAP100	0.851007	-0.0135	0.011449	-0.17702	0.106536	-0.18359
FAAP20	0.084258	-0.13699	0.635533	0.036987	0.212649	0.153893
FAAP24	0.243348	0.323865	0.30407	-0.30031	0.178606	-0.64208
FAN1	0.5256	0.052681	0.000806	-0.29486	0.007179	-0.36737
FANCA	0.009536	0.229599	3.4E-11	0.587951	0.023609	0.339192
FANCB	0.417638	-0.25349	0.633736	0.161247	0.458508	0.393418
FANCC	0.224085	0.133578	0.247685	0.130037	0.902993	-0.02313
FANCD2	0.200247	0.110029	1.37E-14	-0.69652	2.17E-08	-0.82598
FANCE	0.530614	-0.09447	0.032236	0.325687	0.111503	0.400395
FANCF	0.893058	0.017324	0.004733	-0.38592	0.040369	-0.42349
FANCG	0.259455	-0.089	3.1E-26	-0.85069	7.26E-10	-0.78145
FANCI	0.056479	0.122218	0.726696	-0.02321	0.148991	-0.16435
FANCL	0.762748	-0.03855	0.616456	-0.07171	0.792448	-0.05445
FANCM	0.802454	-0.02335	1.38E-46	-1.53567	1.05E-19	-1.53072
FEN1	0.807713	-0.02179	1.74E-08	-0.51682	0.000214	-0.51435
FKBP5	0.050863	0.202848	1.69E-06	0.533018	0.091893	0.31051
FTO	0.040773	0.236782	3.73E-08	0.683319	0.035149	0.426203
GADD45A	0.842525	-0.03632	0.001339	0.569047	0.067341	0.585065
GADD45G	0.320336	-0.1697	0.108007	-0.25015	0.718134	-0.09992
GATA2	0.068035	-0.11687	0.339557	-0.06164	0.851733	0.035242
GEN1	0.491699	0.075089	0.01012	0.294921	0.286871	0.199906
GPS1	0.080644	-0.09847	0.635862	0.026117	0.345153	0.104516
GTF2H1	0.873922	-0.01516	2.15E-06	-0.49395	0.004543	-0.49759

GTF2H2	0.671601	0.071839	0.315277	-0.18214	0.357712	-0.27268
GTF2H3	0.880138	-0.0121	0.013549	-0.21582	0.105584	-0.22277
GTF2H4	0.73158	-0.04579	0.344916	-0.12627	0.647563	-0.09951
GTF2H5	0.456785	-0.1099	0.810692	-0.0382	0.825098	0.052805
H2AC14	0.32852	1.370171	0.479194	1.110104	#N/A	#N/A
H2AC18	0.796642	0.053942	0.000205	0.715478	0.059898	0.637426
H2AC19	0.017525	0.55039	0.000124	0.795062	0.544713	0.227758
H2AC20	0.244923	-0.74227	0.030456	1.532425	0.013499	2.232538
H2AC4	0.139919	-4.13926	0.420956	2.25654	#N/A	#N/A
H2AC6	0.213608	-0.22042	0.031341	0.349461	0.064851	0.549095
H2AC7	0.166669	4.631734	0.343703	-3.22857	#N/A	#N/A
H2AC8	0.465748	0.509429	0.713835	0.253371	0.75565	-0.27504
H2AJ	0.886404	-0.01187	1.17E-13	0.613617	1.73E-06	0.604988
H2AX	0.102806	0.137439	0.873381	0.013385	0.420859	-0.14335
H2AZ1	0.141925	0.07728	2.68E-19	0.488879	0.006899	0.392338
H2AZ2	0.373661	0.062875	0.003171	0.21411	0.40999	0.131802
H2BC11	0.880223	0.138488	0.005628	3.687508	0.012105	3.393581
H2BC12	0.05436	0.386391	0.00017	0.754635	0.326057	0.347818
H2BC13	0.38849	-1.44675	0.467965	1.211275	#N/A	#N/A
H2BC14	0.272019	-2.41591	0.797725	0.527064	#N/A	#N/A
H2BC15	0.793279	0.166398	0.384234	0.538328	0.700215	0.346641
H2BC17	0.763605	0.409321	0.315093	1.469944	#N/A	#N/A
H2BC21	0.655038	-0.09527	0.01362	0.457296	0.11347	0.527548
H2BC3	0.917719	0.311325	0.476967	2.302789	#N/A	#N/A
H2BC4	0.403468	-0.52766	0.675013	0.271344	0.374821	0.772942
H2BC5	0.227323	-0.38593	0.094692	0.501991	0.095393	0.868685
H2BC6	0.776114	0.237904	0.158729	1.190118	0.387267	0.953335
H2BC7	0.643707	0.657992	0.127026	2.062643	0.425378	1.376382
H2BC8	0.729984	0.25995	0.38675	0.592852	0.737631	0.312013
H2BU1	0.313377	0.574221	0.027803	1.152531	0.490782	0.557382
H3-4	0.391007	-2.45784	0.994689	-0.01888	#N/A	#N/A
H4-16	0.194925	0.754786	0.670787	0.231533	0.489871	-0.54158
H4C1	0.256135	1.85449	0.252744	1.811111	#N/A	#N/A
H4C11	0.03243	-1.44839	0.08148	1.170895	0.0074	2.734887
H4C12	0.988242	0.012948	0.483205	0.562385	0.706882	0.445262
H4C14	0.357615	-0.18092	0.01293	0.446076	0.056044	0.588775
H4C15	0.98536	0.011454	0.17816	0.845968	0.1696	0.810058
H4C3	0.781136	-0.50327	0.107321	3.672867	#N/A	#N/A
H4C4	0.965386	-0.08559	0.638162	0.962146	#N/A	#N/A
H4C5	0.081396	-1.81049	0.54825	-0.61389	0.373897	1.137225
H4C8	0.948921	0.022987	0.025978	0.799004	0.175437	0.754257
H4C9	0.969131	-0.13678	0.881669	-0.47391	#N/A	#N/A

HERC2	0.001528	0.212211	0.309606	0.070209	0.152947	-0.16166
HMGB1	0.522278	0.038207	0.814594	0.014729	0.79185	-0.04267
HMGB2	0.000195	0.182813	1.35E-12	0.378427	0.158934	0.176378
HMGN1	0.488164	-0.043	0.845376	-0.01245	0.94596	0.010945
HSD3B1	1.04E-26	15.45229	1E-20	13.48543	0.999586	-1.62767
HSF1	0.484515	0.041474	5.51E-07	-0.2906	0.001116	-0.35194
HUS1	0.206097	-0.19808	0.187716	0.222664	0.137115	0.400351
INO80	0.357064	0.078702	0.359524	-0.08399	0.219019	-0.1828
INO80B	0.248055	0.284454	0.000284	-0.89859	0.003767	-1.19788
INO80C	0.268466	0.292841	0.209567	-0.33746	0.158244	-0.64498
INO80D	0.202683	0.150068	0.758545	-0.0384	0.294657	-0.21205
INO80E	0.282651	-0.08051	7.52E-05	-0.29893	0.060931	-0.23841
ISG15	0.88325	-0.01802	1.49E-13	0.845888	7.38E-06	0.843418
ISY1	0.960855	0.005107	0.32958	0.112718	0.632906	0.088717
KAT5	0.183034	-0.13338	0.000238	-0.38155	0.13042	-0.26777
KDM4A	0.641851	-0.02922	0.996662	0.000269	0.934419	0.009073
KDM4B	0.614552	0.037553	0.000175	0.280081	0.059232	0.222461
KLK2	0.002654	1.111463	1.9E-32	8.036832	2.12E-23	6.847881
KLK3	0.053635	1.388226	1.74E-10	9.664721	2.04E-08	9.037286
KPNA2	0.000195	0.203348	2.63E-06	0.263257	0.785351	0.040625
LATS1	0.037152	0.201936	0.231093	-0.12445	0.036097	-0.34778
LIG1	0.109798	0.14259	0.347403	-0.0846	0.105385	-0.24677
LIG3	0.448299	0.051313	0.350942	0.06835	#N/A	#N/A
LIG4	0.247364	-0.39581	0.062938	-0.68473	0.576527	-0.30807
MAD1L1	0.502341	0.070699	0.742707	0.03603	#N/A	#N/A
MAD2L1	0.07401	0.143155	0.018926	0.201742	0.756723	0.039834
MAD2L2	0.392836	0.076966	5.56E-25	0.933916	2.97E-08	0.837
MAPK8	0.700928	0.038845	0.822775	-0.02519	0.645502	-0.083
MBD1	0.499152	0.066476	0.169147	0.13413	0.774199	0.047224
MBD2	0.444539	-0.06405	0.00841	0.233019	0.045642	0.276948
MBD3	0.13682	-0.14336	0.00059	-0.32823	0.163864	-0.20448
MBD4	0.319047	-0.08802	0.593011	0.051543	0.469543	0.11978
MBD5	0.357736	-0.17026	0.600148	-0.10145	0.881745	0.049781
MCPH1	0.79017	-0.02589	0.012158	-0.26563	0.132822	-0.2586
MCRS1	0.000291	-0.29373	0.124324	-0.12656	0.264455	0.147149
MDC1	0.001185	0.24947	5.39E-05	0.325955	0.634961	0.056727
MDM2	0.380518	0.059148	3.53E-08	-0.39494	4.05E-05	-0.47372
MGMT	0.038319	-0.2267	0.020237	-0.26046	0.777692	-0.05345
MLH1	0.503785	0.053665	1.22E-10	-0.5475	2.33E-06	-0.62085
MLH3	0.075758	0.222109	0.000127	0.499109	0.203971	0.257349
MMP16	0.571044	-0.09481	3.98E-18	-1.47879	9.05E-07	-1.40159
MMS19	0.070237	0.143583	0.182458	-0.10729	0.032665	-0.27082

MNAT1	0.370928	0.113739	0.000588	0.503326	0.094229	0.368996
MPG	0.009442	-0.224	0.507514	-0.0563	0.296391	0.147154
MRE11	0.006856	0.211446	0.000863	0.294132	0.647395	0.06329
MSH2	0.362013	0.076354	0.003374	-0.26542	0.014485	-0.36126
MSH3	0.671461	0.053539	0.08039	-0.23801	0.125467	-0.31086
MSH4	0.748314	0.181535	0.406033	0.486245	0.730378	0.283213
MSH5	0.635737	0.09184	0.510134	-0.13061	0.457474	-0.23749
MSH6	0.127437	-0.11129	0.006196	-0.2096	0.311325	-0.11821
MTOR	0.212258	0.075948	0.082976	0.108739	0.908162	0.012736
MUS81	0.781183	-0.02989	0.00077	0.358374	0.048841	0.370492
MUTYH	0.111037	0.215263	0.033921	0.276305	0.859179	0.038901
N4BP2	6.42E-05	0.569768	0.363852	0.145989	0.061122	-0.4427
NABP2	0.217115	-0.09242	0.574879	-0.04467	0.826132	0.027662
NBN	0.000112	0.300917	0.711303	-0.03134	0.004315	-0.35178
NEIL1	0.301273	0.143995	0.289425	0.146103	0.937892	-0.01758
NEIL2	0.507403	-0.09982	3.06E-07	-0.84105	0.003388	-0.75914
NEIL3	0.000227	0.655198	0.973214	-0.00618	0.029606	-0.68046
NFRKB	0.275248	-0.08509	5.89E-09	-0.46639	0.001181	-0.40064
NHEJ1	0.165811	0.175864	0.220672	0.16574	0.880219	-0.03123
NPLOC4	0.445075	-0.04682	0.117128	0.096569	0.340485	0.123427
NSD2	5.72E-05	0.245961	0.002871	0.187267	0.514477	-0.07877
NTHL1	0.605824	-0.06129	0.584038	-0.06694	0.892023	-0.02558
NUDT1	0.179568	-0.17205	0.040809	0.292902	0.044048	0.444502
NUDT3	0.11724	0.138601	0.284477	-0.09758	0.068908	-0.25586
OGG1	0.849941	-0.01925	0.003223	-0.31236	0.075806	-0.31484
PALB2	0.869961	0.024738	0.287873	-0.17869	0.392731	-0.22427
PARG	0.707877	0.03716	0.326168	-0.10602	0.352702	-0.1642
PARP1	0.97932	-0.00118	0.013993	-0.1147	0.437176	-0.13318
PARP2	0.494116	0.063814	0.396054	-0.08341	0.331158	-0.167
PARP3	0.474043	-0.17917	1.46E-06	-1.21316	0.015059	-1.05298
PARP4	0.348528	0.083842	0.224571	-0.11362	0.144422	-0.2175
PAXIP1	0.330468	-0.09204	0.00431	-0.29942	0.180618	-0.22755
PCLAF	0.066969	0.208634	0.854547	-0.02214	0.188468	-0.2498
PCNA	0.52183	-0.03536	3.96E-06	-0.26921	0.016991	-0.25371
PIAS1	0.498512	0.064646	0.980906	-0.00242	0.599811	-0.08705
PIAS3	0.897111	0.010767	0.28316	0.094253	0.644157	0.063456
PIAS4	0.588385	-0.0545	0.568494	0.058463	0.58939	0.092293
PMS1	0.17222	0.19953	0.002396	-0.46915	0.005145	-0.68827
PMS2	0.142733	-0.11089	0.01113	-0.20011	0.377424	-0.10798
PMS2P1	0.789375	-0.03094	0.051475	-0.24108	0.237499	-0.22783
PMS2P3	0.033405	-0.50338	0.686746	-0.09982	0.353635	0.384704
PMS2P4	0.091449	-0.49908	0.448461	-0.23731	0.642242	0.235913

PMS2P5	0.593737	1.088412	0.692169	-0.825	#N/A	#N/A
PNKP	0.051147	0.280497	0.362782	0.128466	0.483232	-0.17185
POLA1	0.131944	0.196507	0.04052	-0.29254	0.017529	-0.50862
POLA2	0.404737	-0.12295	0.00618	-0.41319	0.196764	-0.31115
POLB	0.14785	0.21532	0.131991	0.233538	0.995869	-0.00134
POLD1	0.841868	-0.01495	0.000307	-0.26433	0.027763	-0.26929
POLD2	0.002035	-0.17158	3.65E-17	-0.48537	0.00452	-0.3336
POLD3	0.795185	-0.02575	1.72E-05	-0.46821	0.009016	-0.4612
POLD4	0.474956	0.142016	0.562677	-0.11574	0.403642	-0.27913
POLE	0.7639	0.020266	0.523032	0.043452	0.978274	0.003186
POLE2	0.193649	0.254511	0.074631	0.37321	0.775819	0.100745
POLE3	0.536082	-0.04284	7.86E-05	-0.28697	0.019755	-0.26358
POLE4	0.080529	-0.26557	0.908175	-0.01808	0.357554	0.228647
POLG	0.246065	-0.08503	0.001672	0.232396	0.011267	0.296779
POLG2	0.306115	0.165468	0.043674	-0.34423	0.061114	-0.53039
POLH	0.007518	0.274128	0.799988	0.026587	0.12453	-0.26743
POLI	0.491355	0.104485	0.98325	-0.00343	0.61499	-0.12486
POLK	0.65275	0.056952	0.231678	0.163699	0.676741	0.087404
POLL	0.346062	0.125801	0.691307	-0.05266	0.346918	-0.19823
POLM	0.583424	-0.0634	0.003055	-0.33838	0.128319	-0.29496
POLN	0.600282	-0.3843	0.240086	-0.86721	0.589423	-0.51841
POLQ	9.41E-06	0.438155	0.007166	0.276063	0.293294	-0.18152
POLR2A	0.486909	0.033081	0.022518	-0.1098	0.30221	-0.16219
POLR2B	0.474478	-0.03896	0.000597	0.199792	0.052152	0.220008
POLR2C	0.102712	-0.1229	3.76E-06	0.367931	0.000117	0.471113
POLR2D	0.833498	-0.01689	0.000502	-0.29138	0.018563	-0.29356
POLR2E	0.029897	-0.1435	0.004007	-0.19256	0.539859	-0.06909
POLR2F	0.100201	-0.17138	0.056897	-0.21489	0.741168	-0.06347
POLR2G	0.079025	-0.15932	3.78E-09	-0.57137	0.006645	-0.43225
POLR2H	0.205668	-0.09627	0.241225	-0.09242	0.902789	-0.01556
POLR2I	0.771979	-0.03397	0.826198	-0.02636	0.964434	-0.00908
POLR2J	0.022892	-0.26995	0.77132	-0.03743	#N/A	#N/A
POLR2K	0.519424	-0.06011	0.13744	0.155396	0.246859	0.196514
POLR2L	0.509016	-0.06304	6.56E-07	-0.48471	0.003126	-0.43989
POT1	0.353892	0.104833	0.109813	0.200418	0.693372	0.075392
PPIE	0.647098	-0.03454	0.030099	-0.17086	0.211285	-0.1562
PPP4C	0.00246	-0.20296	0.159529	-0.09552	0.467831	0.087533
PPP4R2	0.798009	-0.01635	1.16E-07	-0.37369	0.000835	-0.37728
PPP5C	0.896089	0.008005	0.961567	-0.00312	#N/A	#N/A
PRKDC	0.000334	0.191811	1.18E-07	-0.28882	0.000335	-0.50013
PRPF19	0.413143	-0.0497	1.18E-35	-0.77845	1.87E-10	-0.74868
PTTG1	0.471852	0.059963	9.36E-09	0.542925	0.001037	0.46323

RAD1	0.263322	0.108185	0.398408	-0.08629	0.200389	-0.21372
RAD17	0.724673	-0.04193	0.784075	0.034758	0.769819	0.058006
RAD18	0.263459	0.106854	1.43E-07	-0.55687	6.76E-05	-0.68386
RAD21	0.000159	0.19938	0.129265	0.082545	0.357337	-0.13632
RAD23A	0.451942	-0.0457	4.17E-09	-0.36176	0.003966	-0.33539
RAD23B	0.389558	0.048395	0.191957	-0.07701	0.205342	-0.14515
RAD50	0.698408	0.074721	0.075634	-0.34765	0.044385	-0.44008
RAD51	0.337468	0.123275	0.275862	0.14593	0.987081	0.003377
RAD51AP1	0.09422	0.247858	0.843818	0.03209	0.351478	-0.23574
RAD51B	0.817956	-0.08801	0.693201	0.154657	0.699829	0.228065
RAD51C	0.7298	-0.04038	0.906001	0.014713	0.856952	0.035527
RAD51D	0.05502	0.332057	0.058425	-0.33912	0.023138	-0.69164
RAD52	0.028125	0.449793	0.196188	0.277727	#N/A	#N/A
RAD54B	0.153056	0.183916	0.816617	0.031831	0.425946	-0.17178
RAD54L	0.098005	0.16879	0.172701	0.134368	0.754905	-0.05381
RAD9A	0.002029	-0.38035	0.041206	-0.24954	0.594843	0.110961
RAD9B	0.105578	1.4912	0.45985	-0.76752	0.065233	-2.25545
RASSF1	0.041283	-0.20578	0.058429	-0.18706	0.989159	-0.00235
RBBP4	0.050257	0.099301	0.862213	-0.00916	0.37213	-0.12757
RBBP8	0.735717	0.035009	5.48E-12	-0.80173	5.58E-06	-0.85478
RBX1	0.008718	-0.25896	0.460009	0.07755	0.073699	0.317192
RCHY1	0.289168	0.123824	0.010035	-0.33612	0.017522	-0.47902
RDM1	0.183837	0.380972	0.397356	0.253447	0.762581	-0.14729
RECQL4	0.980138	0.001643	0.178806	-0.0846	0.358175	-0.10617
RECQL5	0.801011	0.027305	0.234667	0.128091	0.666368	0.080016
REV1	0.728928	-0.04045	0.801271	0.030825	0.780893	0.052643
REV3L	0.068515	0.193468	0.374714	-0.09907	#N/A	#N/A
RFC1	0.18854	0.091232	0.570168	-0.0428	0.184099	-0.15389
RFC2	0.491748	-0.0498	0.784814	0.02116	0.657118	0.051056
RFC3	0.212102	0.11174	3.68E-06	-0.43759	3.71E-05	-0.56942
RFC4	0.578529	0.051652	0.766469	-0.02923	0.538534	-0.10024
RFC5	0.397553	0.0761	0.805838	-0.02381	0.459541	-0.11857
RHNO1	0.018714	0.273905	0.988553	0.001798	0.12404	-0.2923
RIF1	0.019635	0.18243	0.000221	-0.30399	4.7E-05	-0.50614
RMI1	0.328425	0.102849	0.315606	0.11612	0.972094	-0.00666
RMI2	0.256557	0.179996	0.050635	0.329654	0.630542	0.129055
RNF111	0.726309	0.039113	0.697847	-0.04522	0.583777	-0.10353
RNF168	0.921995	0.009748	0.179002	-0.13858	0.313208	-0.16785
RNF4	0.745984	-0.02259	0.025712	-0.15875	0.177923	-0.15583
RNF8	0.367217	0.122465	0.973892	-0.0047	0.500561	-0.14701
RPA1	0.541701	0.032152	7.58E-11	-0.35562	0.000537	-0.40747
RPA2	0.980464	0.00181	0.31981	0.076237	0.650689	0.054514

RPA3	0.002822	-0.37824	0.553804	0.086356	0.040449	0.445012
RPA4	0.974508	-0.03059	0.080874	-1.84459	0.15898	-1.78965
RPS27A	0.345631	-0.15297	0.672838	0.069078	0.243692	0.205705
RRM1	0.061492	0.107881	2.85E-19	-0.53826	5.07E-09	-0.66568
RRM2	0.041784	0.111554	0.088602	0.095216	0.751137	-0.03632
RRM2B	0.326867	0.095714	0.129999	0.157528	0.80464	0.041941
RTEL1	0.92176	0.018231	0.066825	-0.33725	0.216485	-0.37637
RUVBL1	0.01142	-0.20835	6.25E-06	-0.38844	0.170041	-0.19455
RUVBL2	0.588256	-0.04264	0.485137	0.055497	0.574248	0.078466
SEM1	0.202868	-0.12906	0.562179	-0.06582	0.809021	0.044724
SIRT6	0.257103	-0.15002	0.933951	-0.01099	0.583131	0.118268
SLX1A	0.000288	-0.39161	0.000171	-0.40038	0.879821	-0.02847
SLX1B	0.035292	-0.54699	0.004072	-0.74587	0.62307	-0.22249
SLX4	0.031299	0.22771	0.034783	0.227809	0.909388	-0.02059
SMARCA5	0.25244	-0.0665	4.82E-05	-0.24567	0.081941	-0.19876
SMC1A	0.008337	0.15676	0.260337	0.069977	0.30315	-0.10668
SMC1B	0.162018	-1.12788	0.089863	1.516344	0.013544	2.581204
SMC2	0.02184	0.206676	0.074894	0.177515	0.767122	-0.04879
SMC3	0.046653	0.128296	1.87E-06	-0.33845	2.57E-05	-0.48537
SMC4	5.44E-08	0.276642	7.73E-05	0.210855	0.52838	-0.08519
SMUG1	0.491704	0.096792	0.016796	-0.35266	0.036462	-0.46958
SPIDR	0.980698	0.001796	0.046452	-0.15148	0.139612	-0.17327
SPRTN	0.964616	-0.00551	3.76E-07	-0.6631	#N/A	#N/A
SSRP1	0.732322	0.017406	8.37E-31	-0.62554	1.73E-08	-0.66241
STK3	0.059919	0.212239	9.39E-07	0.577235	0.055971	0.345029
SUMO1	0.375397	-0.07705	0.00022	-0.35675	0.052861	-0.29913
SUMO2	0.156243	-0.08867	0.628169	0.031479	0.500024	0.100692
SUMO3	0.608212	-0.03147	0.738713	-0.02119	0.940723	-0.00961
SUPT16H	0.374935	0.042135	2.64E-05	-0.20925	0.03298	-0.27091
TCEA1	0.012803	0.197615	0.006351	-0.23048	0.001026	-0.44666
TDG	0.543613	0.049808	0.778001	-0.02466	0.461508	-0.09397
TDP1	0.002029	0.298674	0.006236	0.287436	0.851079	-0.03179
TDP2	0.759767	-0.03313	0.074714	0.214126	0.209871	0.227546
TENT4A	0.750694	-0.02059	0.89431	0.008782	0.930168	0.009423
TEP1	0.261718	-0.11845	0.835907	-0.02195	0.675578	0.075883
TERF1	0.428833	0.071084	0.533617	0.057972	0.833661	-0.03367
TERF2	0.718665	-0.04286	0.015393	0.29893	0.110657	0.321296
TERF2IP	0.814434	-0.0179	6.16E-12	0.543993	5.27E-06	0.542115
TERT	0.365616	0.454448	0.136888	0.806167	0.648247	0.330009
TFPT	0.001839	-0.6162	0.298479	0.215317	0.02376	0.811323
TIMELESS	0.041218	0.131581	0.018155	0.156475	0.961893	0.005406
TINF2	0.207988	-0.11032	0.381399	-0.07882	0.939935	0.011066

TIPIN	0.576578	0.111799	0.000147	-0.85568	0.009114	-0.9813
TMPRSS2	5.91E-05	-0.62166	2.46E-97	3.016666	1.53E-49	3.590293
TOP1	0.046108	0.112595	0.000984	-0.19426	0.005235	-0.32582
TOP2A	2.8E-18	0.445191	1.16E-57	0.844061	0.019575	0.379922
TOP2B	0.264999	0.055495	5.01E-08	-0.28317	0.006917	-0.35798
TOP3A	0.066476	0.124323	1.83E-05	-0.29702	7.38E-05	-0.44112
TOP3B	0.358019	-0.09753	0.930098	-0.00927	0.714683	0.067616
TOPBP1	0.017177	0.154537	0.054271	-0.13463	0.006215	-0.30916
TP53	0.432647	-0.05148	6.86E-17	-0.56881	9.52E-07	-0.537
TP53BP1	0.123175	0.111351	0.00052	-0.2612	0.000737	-0.3924
TP53I3	0.101165	0.722351	0.271579	0.486179	0.704561	-0.25439
TP63	0.560678	-1.59164	0.784708	0.552703	#N/A	#N/A
TP73	0.216188	0.51938	0.026704	0.980679	0.498246	0.438481
TRAF6	0.273105	0.169501	0.209609	-0.20469	0.107083	-0.39394
TREX1	0.6206	-0.49605	0.742768	0.329028	0.323101	0.802809
TREX2	0.091603	-1.5277	0.341349	-0.87207	0.595081	0.661718
TRIM25	0.026209	0.160621	0.979004	0.001933	0.129503	-0.17881
TRIM28	0.235767	-0.06	5.74E-09	-0.29523	0.168708	-0.2546
UBA1	0.0304	-0.10085	0.365629	0.042616	0.388567	0.12379
UBA52	0.015314	-0.17907	0.041523	-0.15169	0.955709	0.008951
UBA7	0.221786	1.3933	0.34532	0.94096	0.737159	-0.44499
UBB	0.000291	-0.20355	0.000752	-0.19008	0.976158	-0.00567
UBC	9.13E-09	-0.34808	0.004732	-0.17185	0.413243	0.156922
UBE2A	0.2046	-0.10842	9.67E-06	0.391514	0.000613	0.480303
UBE2B	0.144303	-0.16364	0.146331	0.175389	0.111833	0.319583
UBE2D2	0.061125	-0.14175	0.517081	0.050602	0.153993	0.172273
UBE2D3	0.58572	-0.0352	0.203936	-0.08573	0.564558	-0.06968
UBE2I	0.567374	-0.03654	0.173864	-0.08987	0.523274	-0.0735
UBE2L3	0.58861	-0.03895	0.592392	0.040057	0.612519	0.059525
UBE2N	0.269775	-0.06796	0.003895	-0.19118	0.204204	-0.14249
UBE2T	0.122401	0.163958	0.136156	0.167758	0.935291	-0.01555
UBE2V2	0.844817	0.01709	0.618557	-0.04815	0.5454	-0.08466
UFD1	0.404783	-0.06539	0.007313	0.216739	0.049613	0.262612
UIMC1	0.082862	0.264281	0.375597	-0.1407	0.097319	-0.42434
UNG	0.4806	0.052035	3.99E-10	-0.50023	1.77E-06	-0.57174
UPF1	0.045129	-0.10979	0.000566	-0.18978	#N/A	#N/A
USP1	0.021249	0.146273	0.012364	-0.17009	0.002383	-0.33596
USP10	0.249215	0.087821	0.000127	0.311599	0.087072	0.203948
USP43	0.625	0.117122	0.36124	-0.22702	0.378874	-0.3648
USP45	0.424334	0.132818	0.015684	-0.42012	0.039445	-0.57137
USP7	0.66886	-0.02514	4.51E-07	-0.31	0.008428	-0.30422
UVSSA	0.072722	0.125953	6.38E-06	0.324305	0.112624	0.178322

VCP	0.013848	-0.11959	5.8E-202	-1.50577	2.73E-14	-1.40554
WDR33	0.538225	0.041524	0.914284	-0.00751	0.532454	-0.06905
WDR48	0.641877	-0.04638	3.6E-09	-0.64038	0.000472	-0.61222
WRN	0.6832	0.043876	0.249617	-0.13107	0.305681	-0.19395
WRNIP1	0.296595	-0.0787	0.271451	-0.08421	0.826641	-0.02565
XAB2	0.188294	-0.09693	0.153506	0.105893	0.114035	0.182931
XPA	0.59017	0.096449	0.747889	-0.06332	0.591023	-0.17864
XPC	0.204492	-0.10614	6.99E-06	-0.39931	0.034007	-0.31177
XRCC1	0.872098	-0.0163	0.857246	-0.01899	0.899956	-0.02253
XRCC2	0.54157	0.069552	0.046097	0.251428	0.394812	0.161713
XRCC3	0.208024	-0.11157	1.68E-07	0.449981	0.000127	0.54174
XRCC4	0.985358	0.00396	0.025138	0.566016	0.174352	0.541787
XRCC5	0.434893	-0.04248	1.27E-11	-0.38173	0.010349	-0.35858
XRCC6	0.783456	0.012547	0.508886	-0.03108	0.686532	-0.06338
YAP1	0.025795	0.13077	0.036223	-0.12874	0.010675	-0.27901
YY1	0.25348	0.072361	0.015858	0.158161	0.55105	0.06585
ZNF830	0.010741	-0.35668	0.52182	-0.09722	0.294621	0.239575

Supplementary Table 2: Interaction analysis of differential DDR gene expression of 1245C v/s Empty vector LAPC4, at 0Gy and at 4Gy radiation.

Supplementary Table 3

**Gene List**	**Correlation Coefficient relative to AR**	**P-Value**
**AR**	1	<0.001
**NCOA2**	0.64	<0.001
**EP300**	0.63	<0.001
**CREBBP**	0.61	<0.001
**SPDEF**	0.60	<0.001
**ARID5B**	0.58	<0.001
**LIG3**	0.62	<0.001
**LIG4**	0.51	<0.001
**XRCC5**	0.47	<0.001
**ATR**	0.45	<0.001
**PRKDC**	0.44	<0.001

**MRE11A**	0.43	<0.001
**PARP1**	0.42	<0.001
**ATM**	0.41	<0.001
**RAD51C**	0.39	<0.001
**NBN**	0.36	<0.001
**MSH6**	0.36	<0.001
**XRCC6**	0.36	<0.001
**MSH2**	0.32	<0.001
**RAD54B**	0.30	<0.001
**FANCC**	0.29	<0.001
**USP1**	0.29	<0.001
FANCI	0.10	0.01
XRCC4	0.09	0.013
BRCA1	-0.05	0.12
**BRCA2**	-0.31	<0.001
ACTB	0.21	<0.001
GAPDH	0.48	<0.001
B2M	-0.16	0.68
TBP	0.46	<0.001

Supplementary Table 3: Pearson correlation coefficient of DDR genes with AR. Bolded genes remain significant ($p<0.001$) after multiple correction statistics using Bonferroni correction.
